# Biomechanical strain alters the microRNA cargo of primary myogenic cell-derived extracellular vesicles associated with a differentiation phenotype

**DOI:** 10.3389/fcell.2026.1858881

**Published:** 2026-09-10

**Authors:** Katherine B. Williams, Laura Chubb, Shelby Osburn, Qian Zhang, Bradley Guilliams, Melinda Meyers, Thomas J. LaRocca, Karyn L. Hamilton, Nicole Ehrhart

**Affiliations:** 1 Department of Clinical Sciences, College of Veterinary Medicine and Biomedical Sciences, Colorado State University, Fort Collins, CO, United States; 2 Department of Health and Exercise Science, College of Health and Human Sciences, Colorado State University, Fort Collins, CO, United States; 3 Department of Chemistry, College of Natural Sciences, Colorado State University, Fort Collins, CO, United States; 4 Center for Healthy Aging, Colorado State University, Fort Collins, CO, United States

**Keywords:** differentiation, extracellular vesicles, mechanical strain, mechanobiology, microRNA, myogenesis, myoblast, skeletal muscle

## Abstract

Extracellular vesicles (EVs) are membrane-bound vesicles that regulate intercellular signaling by transporting cellular cargo including RNAs, proteins, and lipids. In recent years, EVs have emerged as promising biologic therapeutics for musculoskeletal repair, recapitulating many of the benefits of mesenchymal stromal cells. However, strategies to enhance the therapeutic potential of EVs remains limited. Here, we investigated how cyclic mechanical strain influences the microRNA (miRNA) cargo and function of EVs produced by primary C57BL/6 murine myogenic cells. Specifically, we aimed to determine how biomechanical strain regimens alter myogenic EV cargo and regulate the transcriptome of recipient myoblasts. We identified miR-222 as a miRNA that was significantly and selectively enriched in low-strain long-duration (LSLD) mechanically strained EVs compared to Static EVs and to high-strain short-duration (HSSD) EVs. Recipient primary myoblasts treated with LSLD EVs displayed distinct transcriptomic changes, characterized by a statistically significant overrepresentation of downregulated predicted target genes associated with miR-222–5p and miR-222–3p at 24 h and 72 h after LSLD EV treatment, respectively. This transcriptomic shift correlated with an increase in Myosin Heavy Chain (MyHC) expression in recipient myoblasts. Together, these findings demonstrate that biomechanical strain regulates the packaging of miRNAs within myogenic EVs, and that LSLD EV delivery is associated with a differentiation-related phenotype in recipient myoblasts. This work provides a foundation for future studies utilizing biomechanical cues to tune EV cargo for potential therapeutic applications in muscle repair and regeneration.

## Introduction

Extracellular vesicles (EVs) are membrane-bound nanoparticles secreted by all cell types that mediate intercellular communication through the transfer of bioactive cargo, including RNAs and proteins (Kalluri and LeBleu, 2020). EV cargo is highly dependent on the cell type and physiological state of the producing cells. For example, cancer cells release EVs that promote proinflammatory and tumorigenic phenotypes (Palmer et al., 2025), whereas stem and progenitor cells, key mediators of tissue regeneration, produce EVs with regenerative properties (Choi et al., 2016; Nakamura et al., 2015; Zhang et al., 2023). Among the types of EV cargo, microRNAs (miRNAs), which are small, non-coding RNAs that regulate gene expression by binding to complementary mRNAs to inhibit their translation and accelerate mRNA degradation (Zhang et al., 2015), play a prominent role in regulating the behavior of recipient cells. Mechanistic studies have demonstrated that EV-contained miRNAs are internalized by recipient cells and contribute to transcriptional changes that influence cell fate (Montecalvo et al., 2012; Valadi et al., 2007).

EVs derived from stem and progenitor cells have emerged as promising candidates for regenerative therapies (Matsuzaka and Yashiro, 2022; Williams and Ehrhart, 2022), including in skeletal muscle repair, where they have been shown to enhance myogenic differentiation, myotube size, and muscle function (Choi et al., 2016; Magarotto et al., 2021; Mullen et al., 2023; Nakamura et al., 2015; Sahu et al., 2021). However, the therapeutic efficacy of EVs has been modest thus far, and optimal culture conditions to produce EVs with robust regenerative activity remain undefined. Moreover, scalable production of therapeutic-grade EVs also remains a challenge (Williams and Ehrhart, 2022).

While donor cell type is a major determinant of EV cargo and function, external signals also influence EV content. Environmental stressors such as inflammation (Hosseinkhani et al., 2018; Xu et al., 2018), radiation (Huber et al., 2024), and cellular senescence (Lee et al., 2023) modulate EV cargo to negatively impact tissue repair. Aging also affects EV biogenesis, as EV and miRNA biogenesis proteins and muscle-specific miRNAs (including miR-1, miR-206, and miR-181a), are altered in aged muscle (Xhuti et al., 2023). Conversely, beneficial stimuli, such as exercise, have been hypothesized to improve the regenerative properties of EVs (Estébanez et al., 2021; Ni et al., 2023; Safdar et al., 2016).

Mechanical loading during exercise varies substantially according to exercise modality (Ozaki et al., 2016). Sustained, lower-magnitude loading is characteristic of endurance exercise, whereas intermittent, higher-magnitude loading occurs during resistance and high-intensity exercise. These loading paradigms activate overlapping but distinct intracellular signaling pathways that regulate skeletal muscle adaptation to exercise (Farup et al., 2012), and have been shown to influence the release of EVs (Frühbeis et al., 2015; Safdar et al., 2016; Whitham et al., 2018). Together, these observations suggest that different patterns of mechanical loading may differentially regulate EV biogenesis and cargo. To model these two distinct forms of mechanical stimulation *in vitro*, we utilized two cyclic strain regimens that were previously developed and validated in our laboratory: a low-strain, long-duration (LSLD) protocol representing sustained mechanical loading, and a high-strain, short-duration (HSSD) protocol representing intermittent, high-intensity loading (Mullen et al., 2023). In our previous study using C2C12 myoblasts, these regimens produced distinct changes in EV production, miRNA cargo, and biological activity (Mullen et al., 2023), providing the rationale for applying the same loading regimens to primary myogenic cells in the current study.

Mechanical strain, an essential component of exercise, stimulates stem cell activity (Ambrosio et al., 2010; Boers et al., 2018; Kadi and Thornell, 2000; Tatsumi, 2010) and improves muscle regeneration (Cezar et al., 2016). Mechanical forces enhance EV production and modulate EV cargo (Guo et al., 2021; Mullen et al., 2023; Thompson and Papoutsakis, 2023). Our previous work demonstrated that mechanical strain applied to C2C12 myoblasts alters the miRNA cargo of EVs to regulate signaling pathways involved in muscle growth, repair, and regeneration, including the MAPK cascade and IGF signaling (Mullen et al., 2023). However, as a transformed and extensively passaged cell line, C2C12 myoblasts may not accurately recapitulate primary myoblast-derived EVs or be suitable for therapeutic applications. For example, late-passage C2C12 cells display impaired differentiation and altered expression of myogenic regulatory factors (Shahini et al., 2018; Sharples et al., 2011). Furthermore, C2C12 myoblasts have been observed to be tumorigenic when transplanted into mice (Wernig et al., 1991). Primary myoblasts, in contrast, offer a more clinically relevant and immunologically compatible EV source for therapeutic development. The use of primary cells in the current study, therefore, represents a key advancement from previous models relying solely on cell lines.

Mechanical strain is a potent regulator of muscle stem cell function and EV biology, but its effects on the miRNA cargo and bioactivity of EVs from primary myogenic cells remain unknown. The present study, therefore, aimed to investigate the hypothesis that cyclic mechanical strain protocols influence the miRNA cargo of primary myogenic progenitor-derived EVs, and that the resulting EVs influence the transcriptome of recipient myoblasts. We highlight miR-222 (Cardinali et al., 2009; 2016; Liu et al., 2024) and miR-148a (Yin et al., 2020; Zhang et al., 2012), miRNAs previously implicated in myogenic differentiation, as two miRNAs selectively enriched in LSLD EVs. We found that LSLD EV treatment was associated with an overrepresentation of predicted targets of miR-222–3p and miR-148a-3p among downregulated genes in recipient cells. These findings provide a hypothesis-generating foundation for future research into the use of biophysical stimuli to modulate EV cargo, offering a starting point for developing specialized culture conditions for future applications in muscle repair.

## Methods

### Primary myogenic cell isolation and myotube differentiation

Hindlimb muscles were harvested from a 13-week-old female C57BL/6 mouse (Jackson Labs). Anesthesia was performed with 3% isoflurane inhalant, followed by euthanasia by cervical dislocation. The mouse was sprayed with 70% ethanol and transferred to a sterile biosafety cabinet. Then skin on the hind leg muscle was cut using sterile Metzenbaum scissors and pulled back to expose the hindlimb muscles. Hindlimb muscles were removed from the mouse using a scalpel and were placed in a sterile Petri dish (Falcon, 353,004). The isolated muscles were briefly submerged in 70% ethanol and rinsed 3 times with sterile Hank’s Balanced Salt Solution (HBSS, Corning, 21–021-CV). Fat and fascia were trimmed from the muscles. The remaining skeletal muscle was then minced into a slurry using a scalpel blade. Five mL of sterile 0.2% Collagenase (Millipore Sigma, C9407) in high glucose (4.5 g/L) Dulbecco’s Modified Eagle Medium (DMEM, Corning, MT10017) was added to the minced muscle tissue. The collagenase muscle mixture was then transferred to a 25 mL Erlenmeyer flask with a stir bar and incubated at 37 °C for 60 min while stirring. Next, the digested muscle was transferred to a 50 mL polypropylene conical tube (Falcon® Corning, 352,098) and the tube was centrifuged using an Allegra™ 6 R centrifuge (Beckman Coulter) at 913 × g (2000 rpm) for 5 min at room temperature (22 °C), equipped with a GH-3.8 swinging-bucket rotor (Beckman Coulter). The pellet in suspension was inverted multiple times and centrifugation was repeated at 913 × g for 5 min at room temperature. The pellet containing digested muscle tissue was then resuspended in 10 mL growth media (containing high glucose (4.5 g/L) DMEM with 20% Fetal Bovine Serum (FBS, SeraPrime, P31016-500) and 1% antibiotic-antimycotic (Corning, 30–004-CI) and mixed gently by pipetting up and down. To identify an optimal substrate for myoblast expansion, muscle tissue suspension was split evenly across two flasks: 5 mL of the suspension was plated on a Matrigel-coated tissue culture-treated T25 flask (Corning, 430,639), and the other 5 mL was plated on a plastic tissue culture-treated T25 flask (Supplementary Figures S1A-B).

After 7 days, the cell culture medium containing suspended pieces of digested muscle tissue from the Matrigel-coated flask was transferred to a 50 mL conical tube. The Matrigel-coated flask was rinsed with 5 mL PBS and this PBS rinse was added to the 50 mL conical tube with cell culture media. The flask, which mainly contained adherent fibroblast-like cells, was discarded. The media with digested muscle was centrifuged using an Allegra™ 6 R centrifuge at 328 *g* (1,200 rpm) for 5 min at room temperature. The media was then removed by vacuum aspiration, and the pellet containing muscle tissue was resuspended in fresh growth media. The muscle tissue was then plated in a fresh Matrigel-coated T75 flask (Corning, 430641U) to allow for a second round of myoblast outgrowth. Small clusters of round cells attached to the suspended tissue (resembling satellite cells) were visible under a microscope (Supplementary Figure S1B).

After two more days, the cell culture medium containing suspended pieces of digested muscle tissue was again transferred to a 15 mL conical tube. The cells in the flask were rinsed with 1X PBS as before, and this PBS rinse was added to the 15 mL conical tube with cell culture media. The flask containing adherent fibroblast-like cells was again discarded. The media was centrifuged at 328 *g* (1,200 rpm) for 5 min at room temperature. The media was then removed by vacuum aspiration, and the pellet containing muscle tissue was resuspended in fresh growth media. The muscle tissue was then plated in a fresh Matrigel-coated T75 flask to allow for a third round of myoblast outgrowth.

After 5 days, all media and remaining suspended tissue was removed by vacuum aspiration. Adherent cells were rinsed with HBSS and were then passaged by detaching the cells using 0.25% trypsin (Corning, 25–053-CI) for 5 min, followed by centrifugation at 447 *g* (1,400 rpm) for 5 min at room temperature. The supernatant was removed, and the cell pellet was resuspended in 12 mL growth media. The cells were seeded on a tissue-culture treated T75 flask and incubated at 37 °C for 1 h to allow fibroblastic cells to adhere to the flask, while myoblasts remained suspended in media. The media containing suspended cells was then transferred to a new Matrigel-coated T75 flask (P1), and the flask containing adherent fibroblasts was discarded.

After 1 day of culture in the Matrigel-coated flask, three distinct adherent cell types could be appreciated: 1) large, stellate-shaped cells with a branched cytoplasm and prominent nucleoli (resembling fibroblasts), 2) small cells with multiple clear, refractive vacuoles characteristic lipid droplets (resembling adipocytes), and 3) small, spindle shaped cells resembling myoblasts (Supplementary Figure S1C). These cells were passaged using 0.25% trypsin and were then seeded on a tissue-culture treated T75 flask and incubated at 37 °C for 1 h to allow fibroblastic cells to adhere. The media containing cells that remained suspended was then transferred to a new Matrigel-coated T75 flask (P2). These remaining cells appeared homogeneous and round, resembling undifferentiated myogenic cells (Supplementary Figure S1D). They were expanded in growth media, and the media was replenished every 4–5 days. When cells were 80% confluent, they were detached using 0.25% trypsin and passaged.

After several passages, the cells appeared to be a homogenous population of spindle-shaped cells, resembling myoblasts (Supplementary Figure S1E). Culturing these cells in serum-depleted differentiation media resulted in differentiation into multinucleated cells (Supplementary Figure S1F) that were observed to spontaneously contract (Supplementary Video S1), characteristic of, and specific for, differentiated myotubes. Finally, immunofluorescence staining of differentiated cells demonstrated positive staining for Myosin Heavy Chain (MyHC, Supplementary Figure S1G), characteristic of differentiated myotubes. A schematic depicting primary myogenic cell isolation is shown in Supplementary Figure S2.

For long-term storage, cells were detached using 0.25% trypsin and centrifuged at 447 *g* (1,400 rpm) for 5 min at room temperature, and the pellet was suspended in freeze media (90% FBS, 10% DMSO (ChemCruz, sc-358801) at one million cells per mL. The cells were aliquoted (1 mL each) in cryovials and stored at −80 °C. After 1 week at −80 °C, cryovials were transferred to liquid nitrogen for long-term storage.

### Matrigel coating

Matrigel (Corning® Matrigel® hESC-qualified Matrix, Cat. 354277) aliquots were thawed at 4 °C and diluted to 1% (v/v) in high glucose (4.5 g/L) DMEM in a sterile 50 mL polypropylene conical tube (Falcon® Corning, 352,098) on ice. Tissue culture-treated T75 and T150 flasks (Corning, 430641U and 430,825) were cooled to 4 °C for 10 min. Matrigel was prepared at 1% v/v in DMEM and added to coat the bottom of each flask (3–6 mL per flask), and flasks were then incubated at 37 °C for 1 h. After 1 h, remaining liquid was aspirated from each flask. Matrigel-coated flasks were stored at 37 °C for up to 1 week prior to cell culture.

### Cell culture

Primary C57BL/6 myogenic cells were cultured in growth media containing high glucose (4.5 g/L) DMEM (Corning, MT-10017-CV), 15% Fetal Bovine Serum (FBS) (SeraPrime, P31016-500), 7.5% heat-inactivated horse serum (HS, Gibco, 16,050–122), 0.4% chick embryo extract (US Biological, C3999), 1% antibiotic-antimycotic (Corning, 30–004-CI), and 2.5 ng/mL bFGF (Peprotech, 100-18B-50UG). Cells were maintained in Matrigel-coated (Corning Matrigel® hESC-qualified Matrix, Cat. 354277) tissue culture treated T75 flasks (Corning, 430641U) at 37 °C in humidified air containing 5% CO_2_ and were not passaged more than 15 times.

To promote differentiation of myoblasts, cells were rinsed with HBSS and fed with differentiation media (DM) containing high glucose (4.5 g/L) DMEM (Corning, MT10017CV or MT10013CV, with or without sodium pyruvate) with 2% EV-depleted Fetal Bovine Serum (FBS, Gibco A27208-01) and 1% antibiotic-antimycotic (Corning, 30–004-CI). For EV isolation, growth media was made with EV-depleted FBS (Gibco, A27208-01) in place of standard FBS to avoid introduction of EVs from FBS within the media (Shelke et al., 2014).

### Immunofluorescence

Primary C57BL/6 myogenic cells (P3) were plated at a density of 20,000 cells per chamber onto 4-chamber glass slides (Lab-Tek, 154,526) in growth media for 24 h, after which the media was switched to DM. After 6 days in DM, cells were washed with PBS and fixed with 4% paraformaldehyde (PFA) (usb, 19,943) for 10 min at room temperature. Following fixation, cells were washed three times with PBS and stored at 4 °C overnight. The following day, cells were rehydrated by rinsing three times with PBS (1–2 min per rinse). Cells were then blocked with mouse blocking buffer (10% mouse serum in 1% bovine serum albumin (BSA, Fisher, BP1605-100)) for 30 min at room temperature. A primary antibody solution was prepared by diluting an anti-Myosin Heavy Chain (MyHC) antibody (clone: MF20, ThermoFisher Scientific; 14–6,503-82) at 1:500 in 0.25% saponin (Sigma, 47,036–50G) in immunofluorescence (IF) buffer (200 uL Triton X-100 (Sigma, T9284-100 ML)), 10 mL 1% BSA, 90 mL PBS containing 0.1% Tween-20(Fisher, PP337-100) (PBS-T)). A 1:500 dilution of the MyHC antibody was added to each well (150 μL each) alongside unstained control wells. Slides were then sealed with parafilm and incubated overnight at 4 °C in the dark. The following day, slides were washed three times with PBS-T. The secondary antibody, Cy3-conjugated AffiniPure donkey anti-mouse IgG (Jackson ImmunoResearch, 715–165-160), was diluted 1:400 in 0.25% saponin (Sigma, 47,036–50G) in IF buffer. Slides were then incubated with the secondary antibody for 30 min at room temperature in the dark, followed by three washes with PBS-T. Cells were then incubated with DAPI (Thermo, D1306) for 10 min to stain nuclei, followed by three additional washes with PBS-T. After allowing slides to dry at room temperature in the dark, samples were coverslipped using Prolong Diamond Anti-fade Mountant (Invitrogen, P36961). Images were captured using an Olympus confocal microscope using Olympus CellSens Software.

### Extracellular vesicle (EV) isolation

At the time of EV collection, the donor culture consisted of a heterogeneous population of differentiating myoblasts and early multinucleated myotubes. Cell culture media from differentiating myogenic cells was collected in sterile 15 mL polypropylene conical tubes (Falcon® Corning, 352,097) for EV isolation. Media was centrifuged at 447 *g* (1,400 rpm) for 5 min at room temperature to pellet large cellular debris. The supernatant was then centrifuged using an AllegraTM 6 R centrifuge in Amicon Ultra-15 filters (Millipore, UFC901024D) with a 10 kDa molecular weight cutoff at 2,800 × g (3,500 rpm) for 45 min at 4 °C to concentrate EVs. The ultrafiltrate (approximately 300 µL) was mixed by pipetting up and down at least 10 times, collected, and then moved to a sterile microcentrifuge tube. The filter was rinsed with approximately 200 µL sterile 1X PBS to collect residual ultrafiltrate, which was added to the microcentrifuge tube to achieve a total volume of 500 µL ultrafiltrate. This concentrated ultrafiltrate was then processed via size-exclusion chromatography (SEC) using qEVoriginal 35 nm size-exclusion chromatography columns (Izon Science) and an automatic fraction collector (Izon Science) to separate small EVs from proteins and larger debris (Supplementary Figure S3A). Briefly, samples were eluted with 1X PBS and ten 0.5 mL fractions were collected. Fractions 1 to 4, identified as the EV-rich, protein-poor fractions (Supplementary Figure S3B), were pooled and sterile filtered using a Foxx Hydrophilic PVDF 0.22 µM membrane filter (Cat # 378–2115-OEM).

EV samples were quantified using a ZetaView® QUATT 4 Nanoparticle Tracking Analyzer (NTA) instrument (Particle Metrix GmbH). Briefly, EV preparations were diluted for an average count of 50–500 particles per frame. The diameter and quantity of EVs were determined using Brownian motion. Size distribution and concentration were taken at 11 positions set to scatter mode using a 488 nm laser; camera CMOS. Data were analyzed using ZetaView® Software version 8.05.12 SP1 (Supplementary Figure S3C). To further concentrate EVs, samples were ultracentrifuged (Optima™ MAX-XP ultracentrifuge, Beckman Coulter) at 110,000 RCF for 1 h and 10 min at 4 °C. The supernatant was pipetted off, and the EV pellet was resuspended in sterile 1X PBS.

### Transmission electron microscopy (TEM)

Primary myogenic cells from a C57BL/6 mouse (P11) were cultured in 25 mL growth media in a T150 Matrigel-coated flask (Corning®, 430,825). Once myoblasts reached approximately 90% confluency, media was removed, and the adherent cells were washed with HBSS. EV-depleted differentiation media containing high glucose (4.5 g/L) DMEM (Corning, MT10017) with 2% EV-depleted Fetal Bovine Serum (FBS, Gibco A27208-1) and 1% Antibiotic-Antimycotic (Corning, 30–004-CI) was then added to the flask. After 24 h, media was collected for EV isolation (as described above in “Extracellular vesicle isolation”). Following size exclusion chromatography (SEC), fractions 1–4 (confirmed to be EV rich and lipoprotein depleted by NTA and protein quantification) were pooled. These pooled EV samples were placed in an ultracentrifuge tube and centrifuged at 100,000 RCF for 70 min at 4 °C using TLA-55 rotor in an Optima™ MAX-XP ultracentrifuge. Sterile 1X PBS (50 µL) was added to the EV pellet and was mixed by pipetting up and down at least 10 times, followed by vortexing for 10 s, to achieve a concentrated EV solution (4.04 × 10^11^ particles per mL, determined by NTA). The EV sample (5 µL) was loaded onto a copper carbon support film 200 mesh (Electron Microscopy Sciences, CF200-CU-50), fixed with 2% paraformaldehyde, and stained with 2% aqueous uranyl acetate. The samples were then examined using a JEOL JEM-2100 F TEM (Supplementary Figures S3D-F). Isolation of EVs and TEM imaging was completed within 5 days after harvesting media from myoblasts. EVs were stored at 4 °C throughout the isolation process.

### Flow cytometry

EVs were isolated from primary C57BL/6 myogenic cells (P10) and were quantified using NTA. A total of 657 μL of EV suspension (containing approximately 5 × 10^9^ EVs) was aliquoted equally into three ultracentrifuge-compatible Eppendorf tubes. Samples were centrifuged at 100,000 RCF for 70 min at 4 °C using a TLA-55 rotor in an Optima™ MAX-XP ultracentrifuge. After centrifugation, 607 μL of supernatant was removed from each tube, leaving 50 μL of PBS with the EV pellet. The EV pellets were resuspended in the remaining PBS by gentle pipetting followed by vortexing for 10 s. Resuspended EVs were stored overnight at 4 °C. Five μL of mouse blocking reagent (10% mouse serum in PBS) was added to each 50 μL EV sample and incubated for 10 min at room temperature to minimize nonspecific antibody binding. The EV samples were then incubated with APC-fluorophore-conjugated antibodies diluted 1:100 in PBS. Two antibodies were used: 1) anti-CD81-APC (BioLegend, 104,909), and 2) an APC-conjugated isotype control (BioLegend, 400,911). The EV samples were incubated with antibodies on ice for 20 min in the dark. An unstained control EV sample was incubated in PBS alone. After antibody staining, 1 mL of PBS was added to each sample. The samples were then ultracentrifuged at 100,000 RCF for 70 min at 4 °C to remove unbound antibodies. Supernatants were discarded, and EV pellets were resuspended in 200 μL PBS by pipetting and brief vortexing. These samples were kept at 4 °C in the dark for 2 h until analysis. For flow cytometry, 150 μL of each sample was then transferred to wells of a 96-well round-bottom plate. The stained EVs were analyzed using a Cytek Aurora four-laser spectral flow cytometer. Analysis and gating were performed using FlowJo™ based on fluorescence intensity and side scatter, with APC + isotype controls used to define positive staining thresholds (Supplementary Figures S3G-I).

### Mechanical strain of primary myogenic cells

Primary C57BL/6 myogenic cells were plated (200,000 per well) in collagen-coated 6-well BioFlex® plates (Flexcell International Corp., BF-3001C COLLAGEN I) in 2 mL growth media per well. When cells were at least 80% confluent and beginning to form visible myotubes, media was removed and the cells were rinsed with HBSS (Corning, 21–021-CV). EV-depleted differentiation media (2 mL) was then added to each well immediately prior to running the mechanical strain protocol.

A FlexCell FX-6000 Tension bioreactor (Flexcell International Corp) was used to apply mechanical strain to cells. Two cyclic tension regimens were created, as described in previously (Mullen et al., 2023). The first regimen subjected cells to cyclic low strain for a long duration (LSLD), with cyclic tension at 0%–15% strain at 0.5 Hz for 24 h. The second regimen subjected cells to a high strain for a short duration (HSSD), of 12%–22% cyclic strain at 1 Hz for 10min, followed by 50 min of rest at 0% strain, which repeated for 24 h. A “static” control group of cells was cultured on BioFlex plates in parallel to the LSLD and HSSD groups and was not subjected to strain. Following 24 h of strain, media was collected for EV isolation.

### Isolation of EVs for small RNA sequencing analysis

Primary C57BL/6 myogenic cells (P4) were cultured in EV-depleted growth media and subjected to mechanical tension using a FlexCell bioreactor as described above. Media was collected from triplicate plates under three strain conditions: LSLD, HSSD, and Static (no strain). EVs were isolated from conditioned media as described above. Following the final ultracentrifugation step to concentrate EVs, PBS was removed by vacuum aspiration and the EV pellet was resuspended in 700 µL QIAzol Lysis Reagent (Qiagen, 79,306), mixed by pipetting and vortexing, and stored at 4 °C for up to 48 h. RNA was isolated from EV samples using the Qiagen miRNeasy Micro Kit (Qiagen, 1,071,023) according to the manufacturer’s instructions. RNA concentration and purity were assessed using a NanoDrop spectrophotometer (Thermo Scientific), and each sample was determined to contain 16–31 ng/μL of EV RNA. Samples were stored at −80 °C until being sent to NovoGene (Sacramento, CA) for small RNA sequencing using single-end 50 bp reads (SE50) at a depth of 20 million reads per sample.

### microRNA analysis

RNA-seq reads were aligned to the *Mus musculus* reference genome using STAR. Reads were mapped and quantified at annotated genomic miRNA loci (precursor/gene level) rather than resolving individual mature 3p and 5p arms. Consequently, mature arm-specific differential expression was not directly measured during the initial transcriptomic alignment. Gene counts mapped to annotated precursor miRNAs were imported into the iDEP2.0 web platform (Ge et al., 2018) for pre-processing and downstream analysis. Genes with a minimum count per million (CPM) of 0.5 were retained, resulting in 493 miRNAs passing this filter. Gene counts were transformed for clustering analysis using EdgeR’s log2 (CPM + c) method, with a pseudo-count of c = 4. Differential miRNA expression analysis was performed using DESeq2, with an adjusted false discovery rate (FDR) threshold of 0.25 and a minimum fold change of 1.5. An FDR threshold of 0.25 was chosen for this exploratory analysis to ensure that subtle shifts in miRNA expression were not missed due to overly conservative correction. DESeq2’s independent filtering was applied to remove low-count genes prior to statistical testing. To compare normalized expression values of specific miRNAs (miR-206 vs. miR-128–1), multiple unpaired t-tests were performed using GraphPad Prism. Volcano plots showing differential miRNAs between EV groups were generated in RStudio using the ggplot2 package.

### mRNA target prediction and gene ontology enrichment analysis

mRNA targets of differential miRNAs were identified using miRDB, an online database for prediction of functional microRNA targets (Chen and Wang, 2020). Because RNA sequencing quantified expression at annotated miRNA loci rather than resolving mature 3p and 5p arms, differential expression was determined at the precursor (gene) level. However, miRNA target prediction requires mature miRNA sequences. Therefore, predicted targets were retrieved separately for each annotated mature arm (3p and 5p) available in miRDB, and enrichment analyses were performed independently for each mature arm miRNA.

For mmu-miR-206–3p, the most highly expressed miRNA across EV samples, 381 targets with a prediction score ≥75 were identified and used for downstream Gene Ontology (GO) analysis. Enrichment of GO analysis was performed using Gorilla (Eden et al., 2009), a web-based tool for identifying and visualizing enriched GO terms. The entire *M. musculus* proteome was used as the background gene set. This analysis resulted in 134 enriched GO Biological Process terms. The GO. db package from Bioconductor was used to identify terminal nodes in RStudio, resulting in 24 significantly enriched terminal GO terms (FDR <0.05).

To evaluate shared functional pathways among the top 10 most highly expressed EV miRNAs, DIANA Tools mirPath v3 (Vlachos et al., 2015) was used to generate a heatmap of enriched GO Biological Process terms based on predicted mRNA targets. For analysis of miRNAs that were commonly upregulated in both HSSD and LSLD EVs compared to Static EVs, target prediction was performed for mmu-miR-301a-3p and mmu-miR-6239 using miRDB. miRDB identified 263 targets with a score ≥80 for mmu-miR-301, and four targets with a score ≥80 for mmu-miR-6239. These combined targets were used as input in GOrilla for GO enrichment analysis, using the *M. musculus* proteome as background set. This analysis identified 193 enriched GO Biological Process terms, and subsequent terminal node filtering using the GO. db package in R Studio identified 41 enriched terminal nodes with FDR <0.05. Due to the low number of predicted targets for miR-6239 (only four targets), separate GO enrichment was not viable for this miRNA, and target prediction was therefore dominated by miR-301a-3p (263 predicted targets).

### EV treatment of myogenic cells for mRNA isolation

Primary C57BL/6 murine myogenic cells (P6) were seeded at a density of 10,000 cells per well in a 24-well plate in growth media. Cells were allowed to adhere and expand for 5 days, after which the wells were rinsed with Hanks Balanced Sald Solution (HBSS) (Corning, MT10021CV), and 1 mL of EV-depleted growth medium (see above) was added to each well. Wells were then treated in triplicate with 1 × 10^10^ EVs per well derived from low-strain long-duration (LSLD), high-strain short-duration (HSSD), or static myoblasts. Control wells received the PBS vehicle alone. An EV suspension containing 1 × 10^10^ EVs or PBS vehicle alone (32 µL) was added to each well to ensure consistent EV concentration across treatment groups. RNA was collected at two timepoints post-treatment: 24 h and 72 h. At 24 h post treatment, media was aspirated from three wells per treatment group, and cells were rinsed with sterile 1X PBS. Trizol reagent (Invitrogen™, 15,596,018), 700 μL, was added to each well and cell lysates were mixed by pipetting, collected, and stored at −80 °C. At 72 h post-treatment, the same Trizol collection procedure was repeated for the remaining triplicate wells for each treatment condition. Total RNA was extracted using the Qiagen miRNeasy Micro Kit according to the manufacturer’s instructions. RNA concentration and purity were assessed using a NanoDrop (Thermo Scientific) spectrophotometer. Samples were stored at −80 °C until being sent to NovoGene (Sacramento, CA) for bulk RNA sequencing. Samples were sequenced at a depth of approximately 9 Gb per sample, generating approximately 30 million paired-end 150 bp read pairs per sample.

### mRNA analysis

RNA-seq reads were aligned to the *M. musculus* reference genome using STAR. Gene count data were imported into the iDEP.96 web platform (Ge et al., 2018) for pre-processing and downstream analysis. Genes with a minimum count per million (CPM) of 0.5 were retained, resulting in 16,266 genes passing the filter. Gene counts were transformed for clustering analysis using EdgeR’s log2(CPM + c) method, with a pseudo-count of c = 4. A heatmap displaying hierarchical clustering of the 1,000 most variable genes was generated in iDEP.96. Principal Component Analysis (PCA) was also performed using iDEP.96 to visualize global transcriptomic differences between treatment groups.

Differential gene expression analysis was performed using DESeq2, with an adjusted false discovery rate (FDR) threshold of 0.1 and a minimum fold change of 1.5. Enriched GO Biological Process terms for differentially expressed genes were subsequently identified using the iDEP.96 web platform. Area-proportional Venn diagrams showing commonly upregulated or downregulated genes across pairwise comparisons were generated using the eulerr package in R Studio.

### Predicted miRNA target enrichment analysis

To evaluate whether mechanically regulated EV-associated miRNAs were associated with the transcriptomic response observed in recipient primary myoblasts, enrichment analyses were performed to determine whether predicted miRNA target genes were overrepresented among genes downregulated following treatment with LSLD or HSSD EVs. A targeted enrichment analysis was performed for each mature arm of the ten candidate miRNAs enriched in LSLD EVs relative to Static EVs. Predicted mRNA targets for each candidate miRNA were obtained from mature-arm target lists derived from miRDB (Chen and Wang, 2020), and all predicted targets were retained.

Differential gene expression results from recipient myoblast RNA sequencing were analyzed separately for the 24-h and 72-h time points. Downregulated genes were defined as those with a false discovery rate (FDR) < 0.10 and an absolute fold change ≥1.5 (equivalent to an absolute log_2_ fold change ≥0.585).

Enrichment analyses were performed separately for each time point using the corresponding DESeq2 gene universe as the statistical background. The gene universe consisted of all genes retained following DESeq2 preprocessing and filtering that were eligible for differential expression testing. Prior to analysis, both the downregulated gene lists and the predicted miRNA target lists were restricted to genes present in the corresponding DESeq2 gene universe. Genes not present in the background universe were excluded from the enrichment analysis because they were not eligible for differential expression testing and therefore could not appropriately contribute to the contingency table. Duplicate gene identifiers and missing values were removed before analysis so that each gene was counted only once.

For each miRNA, enrichment of predicted targets among downregulated genes was evaluated using a one-sided Fisher’s exact test (alternative = “greater”), testing whether predicted miRNA targets occurred more frequently among downregulated genes than expected by chance. For each miRNA, a 2 × 2 contingency table was constructed comparing predicted target status with differential expression status. Expected overlap between predicted target genes and downregulated genes was calculated from the sizes of the predicted target list, the downregulated gene set, and the background gene set. Fold enrichment was calculated as the ratio of observed overlap to expected overlap. Odds ratios and corresponding 95% confidence intervals were calculated to quantify the magnitude of enrichment (Supplementary Tables S1, 2).

Statistical significance was defined as a Fisher’s exact test p-value <0.05. Results for each miRNA included the number of predicted target genes, observed overlap, expected overlap, fold enrichment, odds ratio with 95% confidence interval, and p-value. Bubble plots summarizing enrichment analyses were generated in R using the ggplot2 package, with fold enrichment plotted on the x-axis, observed overlap represented by bubble size, and p-value represented by bubble color. MiRNAs meeting the predefined significance threshold (P < 0.05) were highlighted using thick outlines.

### Deuterium enrichment DNA synthesis assay

C57BL6 primary myogenic cells (20,000 cells from passage 14) were plated per well in 24-well tissue-culture plates (VWR, 10,861–700) in growth media. Cells were incubated overnight at 37 °C to allow adherence. The following day, when cells were approximately 20% confluent, growth media was removed by vacuum aspiration and adherent cells were rinsed with sterile 1X PBS. EV-depleted growth media enriched with 10% deuterium oxide (D_2_O) was then added to each well. Wells were treated in triplicate with 1e10 EV s isolated from Static, HSSD, or LSLD primary myoblasts. Control wells received an equal volume of the sterile PBS vehicle. Samples were collected at two time points: 24 h and 48 h post-treatment. At each of these timepoints, 1 mL of media was collected from each plate for assessment of precursor enrichment. The remaining media was removed by aspiration, and cells were rinsed with 1X PBS. The cells were then scraped in 75 µL of the isolation buffer (100 mM KCl, 40 mM Tris HCl, 10 mM Tris Base, 5 mM MgCl2, 1 mM EDTA, 1 mM ATP, pH = 7.5) with phosphatase and protease inhibitors (HALT, Thermo Scientific, Cat# 78442) using a sterile P1000 pipette tip. Cell lysates and media samples were stored at −80 °C until subsequent analysis by gas chromatography-mass spectrometry (GC-MS).

The rate of DNA synthesis was determined by the detection of deuterium incorporation into purine deoxyribose (dR) of DNA as described previously (Bruns et al., 2018; Wolff et al., 2020). Briefly, 20 µL of cells in isolation buffer were sonicated to release DNA before hydrolysis overnight at 37 °C with nuclease S1 and potato acid phosphatase. For derivation, hydrolysates were reacted with pentafluorobenzyl hydroxylamine and acetic acid, then acetylated with acetic anhydride and *1*-methylimidazole. The derivatives were extracted by dichloromethane, dried, resuspended in ethyl acetate, and then analyzed by a 7890 B gas chromatograph coupled to a 7,010 mass spectrometer with a DB-17 column (all from Agilent) under negative chemical ionization using helium as carrier and methane as the reagent gas. To assess culture media D_2_O enrichment level, water was extracted from 125 μL of media placed into the inner well of an O-ring screw-on cap, and placed inverted on a heat block overnight. Next, 2 μL of 10 M NaOH and 20 μL of acetone were added to the water samples and capped immediately. After mixing briefly, the samples were incubated at room temperature overnight. The subsequent products were extracted by adding 200 μL hexane and transferring the organic layer through anhydrous Na_2_SO_4_ into gas chromatography vials. The final products were analyzed via EI mode on 7890 A gas chromatograph coupled to a 5975C mass spectrometer with a DB-17 column (all from Agilent). The fraction new was calculated by dividing DNA enrichment by the media enrichment with MIDA adjustment (Busch et al., 2007), and divided by time (FSR, %/h). To assess statistical differences in DNA synthesis, all experimental groups (LSLD EV, HSSD EV, Static EV, and PBS vehicle control) were compared to one another using a one-way ANOVA followed by Tukey’s *post hoc* multiple comparisons test.

### Myogenic differentiation assay

Primary C57BL/6 myogenic cells (P14) were plated at a density of 20,000 cells per chamber onto 2-chamber (Permanox) glass slides (Thermo Scientific Nunc, 177,429) in growth media for 5 days, until they were approaching 100% confluency but not yet visibly differentiating into myotubes. At that time, the media was removed and the cells in wells rinsed with HBSS. EV-depleted growth media, 1 mL, was then added to each well along with 9.5e9 EVs from either Static, LSLD, or HSSD primary myoblasts, in triplicate. Control wells received an equal volume of the sterile PBS vehicle.

Two days after treatment, cells were washed with phosphate-buffered saline (PBS) and fixed with 4% paraformaldehyde (PFA, usb 19,943) for 10 min at room temperature. Following fixation, cells were washed three times with PBS and stored at 4 °C overnight. The following day, cells were rehydrated by rinsing three times with PBS-T (1–2 min per rinse). Cells were then blocked with donkey blocking buffer (10% donkey serum (Jackson ImmunoResearch, 017–000-121) in 1% bovine serum albumin (BSA) (Fisher, BP1605-100) for 30 min at room temperature. A primary antibody solution was prepared by diluting anti-Myosin Heavy Chain (MyHC) antibody (clone: MF20, ThermoFisher Scientific; 14–6,503-82) at 1:500 in 0.25% saponin (Sigma, 47,036–50G) in immunofluorescence (IF) buffer (PBS +0.1% Tween 20 (Fisher, PP337-100) + 0.2% Triton X-100 (Sigma, T9284-100 ML) + 10% of a 1% BSA in DI water)). The MyHC antibody was added at 150 μL to each well, and PBS was added to unstained control wells. Slides were then sealed with paraffin film and incubated overnight at 4 °C in the dark. The following day, slides were washed three times with PBS-T. The secondary antibody, Cy3-conjugated AffiniPure donkey anti-mouse IgG (Jackson ImmunoResearch, 715–165-160), was diluted 1:400 in 0.25% saponin in IF buffer. Slides were then incubated with the secondary antibody for 30 min at room temperature in the dark, followed by three washes with PBS-T. Cells were then incubated with DAPI (Thermo, D1306) for 10 min to stain nuclei, followed by three additional washes with PBS-T. After allowing slides to dry at room temperature in the dark, samples were mounted with Diamond Antifade Mountant (Invitrogen, P36961) and coverslipped.

Images were captured on an Olympus confocal microscope using Olympus CellSens Software. Three wells were imaged per treatment group and eight images were taken within each well in a 3 × 3 automated grid pattern. ImageJ was used to apply a mask with a threshold set to include MyHC + areas of each image. The percent area of each image above this threshold (corresponding to MyHC positive areas) was quantified, and the values of each of the eight images taken per well was averaged for each replicate. A one-way analysis of variance (ANOVA) followed by Tukey’s multiple comparisons test was used to statistically compare the average percent area of positivity for MyHC between treatment groups (n = 3 replicates per group) in GraphPad Prism. Images provided in the manuscript were pseudo-colored with the “Red Hot” Lookup Table for MyHC staining, and the “Blue” Lookup Table for DAPI staining, using ImageJ.

### Ethics approval

Animal procedures were conducted in compliance with the Animal Welfare Act and approved by the Institutional Animal Care and Use Committee at Colorado State University (IACUC) #1102. A 13-week-old female C57BL/6 mouse purchased from Jackson Labs was used for primary skeletal muscle harvest and subsequent muscle cell isolation. The mouse was housed in an IACUC-approved facility at the Veterinary Teaching Hospital at Colorado State University and was cared for by trained staff of the Laboratory Animal Resource Center. The mouse was provided with water and rodent chow *ad libitum*. The mouse was humanely euthanized by rapid cervical dislocation under 3% isoflurane inhalant anesthesia, as recommended by the American Veterinary Medical Association. All authors understand the ethical principles under which the journal operates and agree that this study complies with this animal ethics checklist.

### Statistical analysis

For direct comparisons between two experimental groups, unpaired two-tailed Student's t-tests were performed. This included the comparison of normalized expression values between specific highly abundant miRNAs (miR-206 vs. miR-128–1) and the comparison of total EV production across mechanical strain conditions.

For analyses involving three or more independent treatment groups, differences were analyzed utilizing a one-way ANOVA followed by Tukey’s multiple comparisons test to determine specific inter-group differences. This included evaluation of differences in the fraction of new DNA synthesized per hour (D_2_O incorporation assay), cell confluence over multiple timepoints, and the average percent area positive for MyHC in myogenic differentiation assays. For all *in vitro* assays, a *p*-value of <0.05 was considered statistically significant. Statistical analyses and graphical representations were performed using GraphPad Prism.

## Results

### Primary C57BL/6 myogenic cells produce CD81^+^ extracellular vesicles

Primary cells were isolated from C57BL/6 mouse hindlimb muscles and were expanded in culture. Myogenic lineage was validated by visualization of multinucleated cells (Supplementary Figures S1F), positive immunostaining for MyHC (Supplementary Figures S1G), and observation of spontaneous contraction in culture (Supplementary Video S1). EVs were then isolated from primary myogenic culture cell media using a combination of ultrafiltration, size exclusion chromatography, and ultracentrifugation (Supplementary Figures S3A-B). Based on NTA, primary myogenic EVs had a median diameter of 113 nm ± 56 nm, which is within the expected size range of small EVs (Supplementary Figures S3C).

Scanning transmission electron microscopy (STEM) and transmission electron microscopy (TEM) identified electron-dense vesicles with distinct spherical morphology, characteristic of small EVs (Supplementary Figures S3D-F). Finally, flow cytometry was used to detect CD81, an exosome-specific surface marker, within myogenic cell-derived EVs, demonstrating that 48% of EVs stained positive for CD81 (Supplementary Figures S3G-I). Total EV production was influenced by mechanical strain, with the HSSD regimen resulting in a 2.5-fold increase in EV production by primary myogenic cells (Supplementary Figure S4).

### Mechanical strain of primary myogenic cells results in differential expression of miRNAs packaged into EVs

**FIGURE 1:**
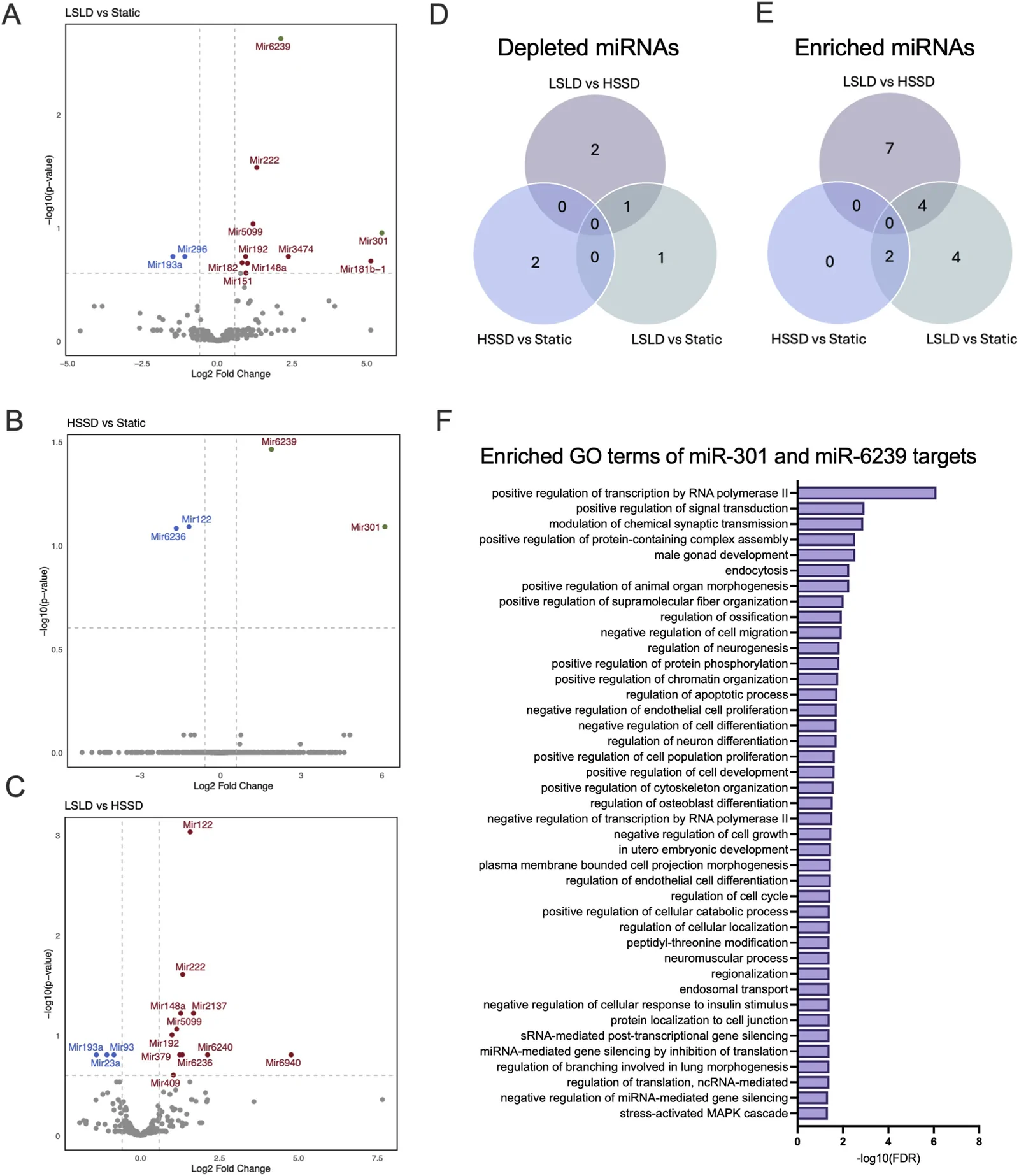
Differential miRNAs within EVs produced by differentiating myoblasts in response to mechanical strain. **(A–C)** Volcano plots of significantly enriched and significantly depleted miRNAs in **(A)** LSLD vs. Static EVs **(B)** HSSD vs. Static EVs, and **(C)** LSLD vs. HSSD EVs. FDR <0.25 and an absolute fold change >1.5 indicates significance. Significantly depleted miRNAs are labeled in blue and significantly enriched miRNAs are labeled in red. MiRNAs that were commonly enriched in both LSLD and HSSD EVs compared to Static are labeled in green. **(D,E)** Venn diagram depicting unique and shared differentially **(D)** depleted and **(E)** enriched miRNAs between strain conditions. **(F)** Terminal nodes of enriched gene ontology (GO) terms of the predicted mRNA targets of miR-301 and miR-6239, which were enriched in both LSLD and HSSD EVs compared to Static EVs.

Small RNA sequencing was performed on EVs isolated from Static (unstrained), HSSD, and LSLD mechanical strain conditions applied to primary myogenic cells to determine how mechanical strain influences the small RNA content of myogenic EVs. Differential expression analysis was performed on miRNAs to identify differences in EV-associated miRNAs between strain conditions. Between LSLD EVs and Static EVs, 10 miRNAs were enriched, and two miRNAs were depleted (Figures 1A,D,E; Table 1) in LSLD EVs. HSSD EVs showed fewer changes in miRNA expression compared to Static EVs: two miRNAs were enriched, and two miRNAs were depleted (Figures 1B,D,E; Table 1). LSLD EVs also showed differences in miRNA expression compared to HSSD EVs: 11 miRNAs were enriched, and three miRNAs were depleted in LSLD EVs compared to HSSD EVs (Figures 1C–E; Table 1).

**TABLE 1:** Differential miRNAs between Static EVs, HSSD EVs, and LSLD EVs. * = FDR <0.05, ** = FDR <0.01, *** = FDR <0.001.

LSLD vs. Static			HSSD vs. Static			LSLD vs. HSSD		
miRNA	Log2FC	FDR	miRNA	Log2FC	FDR	miRNA	Log2FC	FDR
*miR-6239*	2.1263	0.0021**	*miR-6239*	1.8936	0.0344*	*miR-122*	1.5650	0.0009***
*miR-222*	1.3248	0.0291*	*miR-301*	6.1080	0.0812	*miR-222*	1.3299	0.0244*
*miR-5099*	1.1984	0.0914	*miR-122*	−1.1766	0.0812	*miR-148a*	1.2670	0.0598
*miR-301*	5.5009	0.1100	*miR-6236*	−1.6511	0.0827	*miR-2137*	1.6733	0.0598
*miR-192*	0.9483	0.1779	​	​	​	*miR-5099*	1.1385	0.0859
*miR-296*	−1.0816	0.1779	​	​	​	*miR-192*	0.9893	0.0982
*miR-193a*	−1.4804	0.1779	​	​	​	*miR-6236*	1.3175	0.1552
*miR-3474*	2.3765	0.1779	​	​	​	*miR-379*	1.2365	0.1552
*miR-181b-1*	5.1339	0.1951	​	​	​	*miR-193a*	−1.3983	0.1552
*miR-182*	0.8309	0.2018	​	​	​	*miR-6940*	4.7659	0.1552
*miR-148a*	1.0125	0.2043	​	​	​	*miR-93*	−0.8417	0.1552
*miR-151*	0.9603	0.2491	​	​	​	*miR-6240*	2.1164	0.1552
​	​	​	​	​	​	*miR-23a*	−1.0670	0.1552
​	​	​	​	​	​	*miR-409*	1.0329	0.2481

The most abundant miRNAs, in primary myogenic EVs, including miR-206 and miR-128, are not significantly altered by mechanical strain. miRNAs with FDR < 0.05 should be bolded.

Notably, differential expression analysis revealed that miR-222 was highly and selectively enriched in LSLD EVs compared to both Static EVs (Log2FC = 1.325, FDR = 0.0291) and to HSSD EVs (Log2FC = 1.330, FDR = 0.0244) (Table 1; Figures 1A,C). Similarly, miR-148a demonstrated a pattern of LSLD-specific cargo enrichment, exhibiting upregulation in LSLD EVs compared to HSSD EVs (Log2FC = 1.267, FDR = 0.0598) and compared to Static EVs (Log2FC = 1.013, FDR = 0.2043) (Table 1; Figures 1A,C). Conversely, miR-301 (LSLD vs. Static: Log2FC = 5.501, FDR = 0.1100; HSSD vs. Static: Log2FC = 6.108, FDR = 0.0812) and miR-6239 (LSLD vs. Static: Log2FC = 2.126, FDR = 0.0021; HSSD vs. Static: Log2FC = 1.894, FDR = 0.0344) were identified as broadly strain-regulated species commonly packaged in response to physical stretch, enriched in both LSLD EVs and in HSSD EVs compared to Static EVs.

To investigate potential functional roles of miR-301 and miR-6239, which were commonly enriched in both LSLD and HSSD EVs compared to Static EVs, GO enrichment analysis was performed on the predicted mRNA targets of miR-301 and miR-6239. Enriched biological processes included terms related to regulation of transcription and translation, neuromuscular processes, and cell differentiation (Figure 1F).

The majority of the sequencing reads in all EV samples were annotated as miRNAs, with lower amounts annotated as coding RNAs, lncRNAs, and snoRNAs (Supplementary Figure S5A), indicating that miRNAs are the predominant small RNA species packaged into myoblast EVs regardless of mechanical strain.

The top 10 most abundant miRNAs in EV samples from each strain condition were the same, suggesting that mechanical strain does not drastically alter the levels of the predominant miRNAs packed into myogenic EVs (Supplementary Figure S5B). In particular, miR-206 was most abundant in all samples, with significantly higher levels than the second most abundant miRNA, miR-128–1 (p = 0.0004, unpaired t-test), suggesting that miR-206 is preferentially packaged into myogenic cell-derived EVs regardless of strain protocol (Supplementary Figure S5B).

To investigate potential biological roles of miR-206, GO enrichment analysis was performed on the predicted mRNA targets of this miRNA. Significantly enriched GO biological processes included terms related to tissue repair and remodeling, particularly of muscle and nervous tissue (i.e., muscle cell differentiation, cell proliferation, cell migration, cytoskeleton organization, regulation of neurogenesis, Supplementary Figure S5C). A broader GO enrichment analysis of the top 10 miRNAs in all samples identified enrichment of shared GO terms related to transcriptional regulation, cytoskeleton organization, and cell dynamics (i.e., motility, differentiation, and proliferation), demonstrating shared functional targets of the predominant miRNAs within myogenic EVs (Supplementary Figure S5D). While many of these enriched terms are relatively generic and thus commonly observed in enrichment analyses, their reoccurrence in the context of myogenic EVs suggests a role for these miRNAs in muscle cell differentiation and remodeling.

### EVs derived from myogenic cells exposed to mechanical strain affect gene expression in recipient cells by 24 h after EV exposure

**FIGURE 2:**
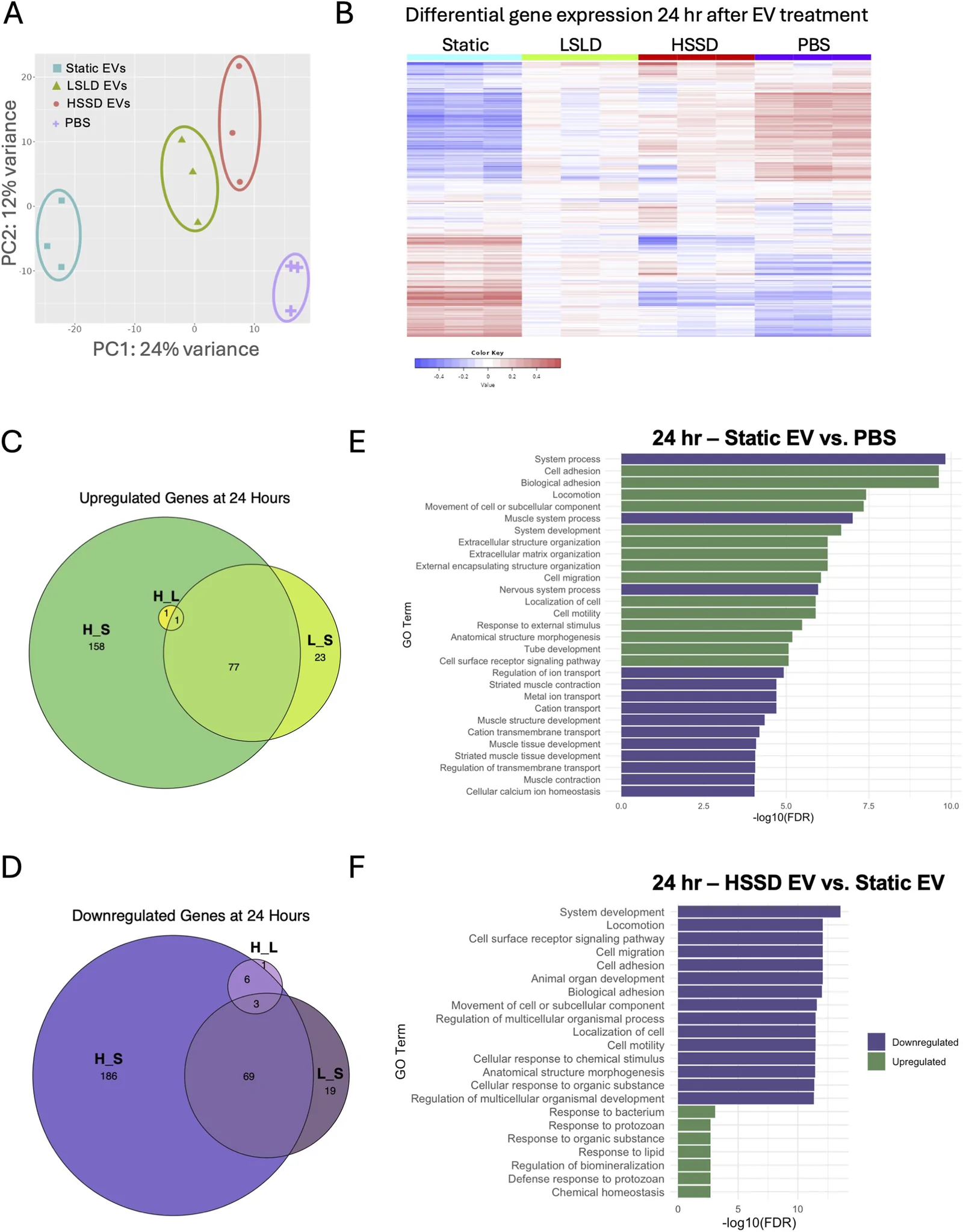
Mechanically strained myogenic cell-derived EVs induce distinct transcriptomic signatures in recipient myoblasts 24 h after EV exposure. **(A)** Principal component analysis (PCA) showing clustering of transcriptomic signatures of recipient myoblasts 24 h after treatment with EVs derived from Static, LSLD, or HSSD myoblasts, or PBS controls. **(B)** Heatmap showing differentially expressed genes across treatment groups, with samples clustered by expression of the top 1,000 most variable genes based on mRNA read counts. Red indicates relatively higher expression; blue indicates relatively lower expression. Blue bar = Static EV-treated; Purple bar = PBS treated; Red bar = HSSD EV-treated; Green bar = LSLD EV-treated **(C)** Venn diagram comparing upregulated genes in three pairwise comparisons: HSSD vs. Static EV-treated myoblasts (H_S), LSLD vs. Static EV-treated myoblasts (L_S), and HSSD vs. LSLD EV-treated myoblasts (H_L). **(D)** Venn diagram comparing downregulated genes in three pairwise comparisons: HSSD vs. Static EV-treated myoblasts (H_S), LSLD vs. Static EV-treated myoblasts (L_S), and HSSD vs. LSLD EV-treated myoblasts (H_L). **(E)** Gene ontology (GO) enrichment analysis of Biological Process terms for Static EV-treated myoblasts vs. PBS vehicle-treated controls 24 h after EV exposure. **(F)** GO enrichment analysis of Biological Process terms for HSSD EV-treated myoblasts vs. Static EV-treated myoblasts 24 h after treatment. Purple shows downregulated GO terms and green shows upregulated GO terms. GO enrichment p-values were corrected for multiple hypothesis testing using the Benjamini–Hochberg false discovery rate (FDR) procedure, and significance is presented as -log_10_(FDR). GO terms with FDR <0.05 were considered statistically significant.

Twenty-four hours after EV exposure, recipient myogenic cells (n = 3 replicates per treatment group) were harvested for mRNA sequencing. Principal component analysis (PCA) revealed that replicates clustered by treatment group, indicating distinct gene expression signatures associated with each treatment condition (Figure 2A). A heatmap of the top 1,000 most variable genes further highlighted treatment-dependent expression patterns in myogenic cells that received EVs, with the most pronounced differences in gene expression occurring between Static EV-treated cells and the PBS vehicle control (Figure 2B). Notably, myogenic cells treated with LSLD EVs or with HSSD EVs shared similar transcriptomic profiles, which clustered distinctly away from Static EV-treated cells, suggesting that EVs derived from strained myogenic cells, regardless of strain protocol, induce comparable transcriptional responses 24-h post-treatment.

Differential gene expression analysis was then performed between treatment groups. A total of 158 genes were uniquely upregulated in HSSD EV-treated cells compared to Static EV-treated cells, while 77 genes were commonly upregulated in both HSSD EV vs. Static EV-treated cells and in LSLD EV vs. Static EV-treated cells, suggesting a common gene expression pattern following treatment with either HSSD EVs or LSLD EVs compared to treatment with Static EVs. A total of 23 genes were uniquely upregulated in LSLD EV vs. Static EV-treated cells, indicating that some genes were uniquely regulated by LSLD EV treatment. Only two genes were differentially upregulated with HSSD EV treatment compared to LSLD EV treatment, indicating that EVs from both strain conditions induce similar gene expression patterns 24 h post-treatment (Figure 2C).

A similar trend was observed in downregulated genes. A total of 186 genes were uniquely downregulated in HSSD EV compared to Static EV-treated cells, and 69 genes were commonly downregulated in both HSSD EV vs. Static EV-treated cells, and in LSLD EV vs. Static EV-treated cells. A total of 19 genes were uniquely downregulated in LSLD EV vs. Static EV-treated cells. Ten genes were differentially downregulated with HSSD EV treatment compared to LSLD EV treatment (Figure 2D).

Differentially expressed GO terms were evaluated between Static EV vs. PBS vehicle-treated controls, because these two groups had the greatest number of differentially expressed genes. Compared to PBS vehicle-treated controls, cells treated with Static EVs displayed upregulated GO terms related to cell movement, structural organization, and cell signaling (i.e., cell adhesion, ECM organization, cell migration, cell surface receptor signaling), and downregulated GO terms related to myogenic maturation and ion transport (striated muscle contraction, muscle structure development, calcium ion homeostasis). Overall, these data suggest that Static EV treatment is associated with a gene expression profile consistent with an earlier, less differentiated myoblast phenotype, characterized by an enrichment of pathways related to cell motility rather than myogenic maturation (Figure 2E).

Next, differentially expressed GO terms between HSSD EV vs. Static myoblasts were analyzed to identify differences in EV treatment related to mechanical strain. The upregulated GO terms in cells treated with HSSD EVs compared to Static EVs, including response to an organic substance, a protozoan, and a bacterium, are associated with a transcriptomic signature related to immune and inflammatory signaling. The downregulated GO terms following treatment with HSSD EVs compared to Static EVs included cell motility, migration, and adhesion (Figure 2F), indicating a transcriptomic signature associated with reduced cell migration. Overall, this GO analysis identified biological pathways, including inflammation and migration, that may be impacted by EVs generated by mechanically strained myogenic cells.

### EVs derived from myogenic cells exposed to mechanical strain affect gene expression in recipient cells 72 h after EV exposure

**FIGURE 3:**
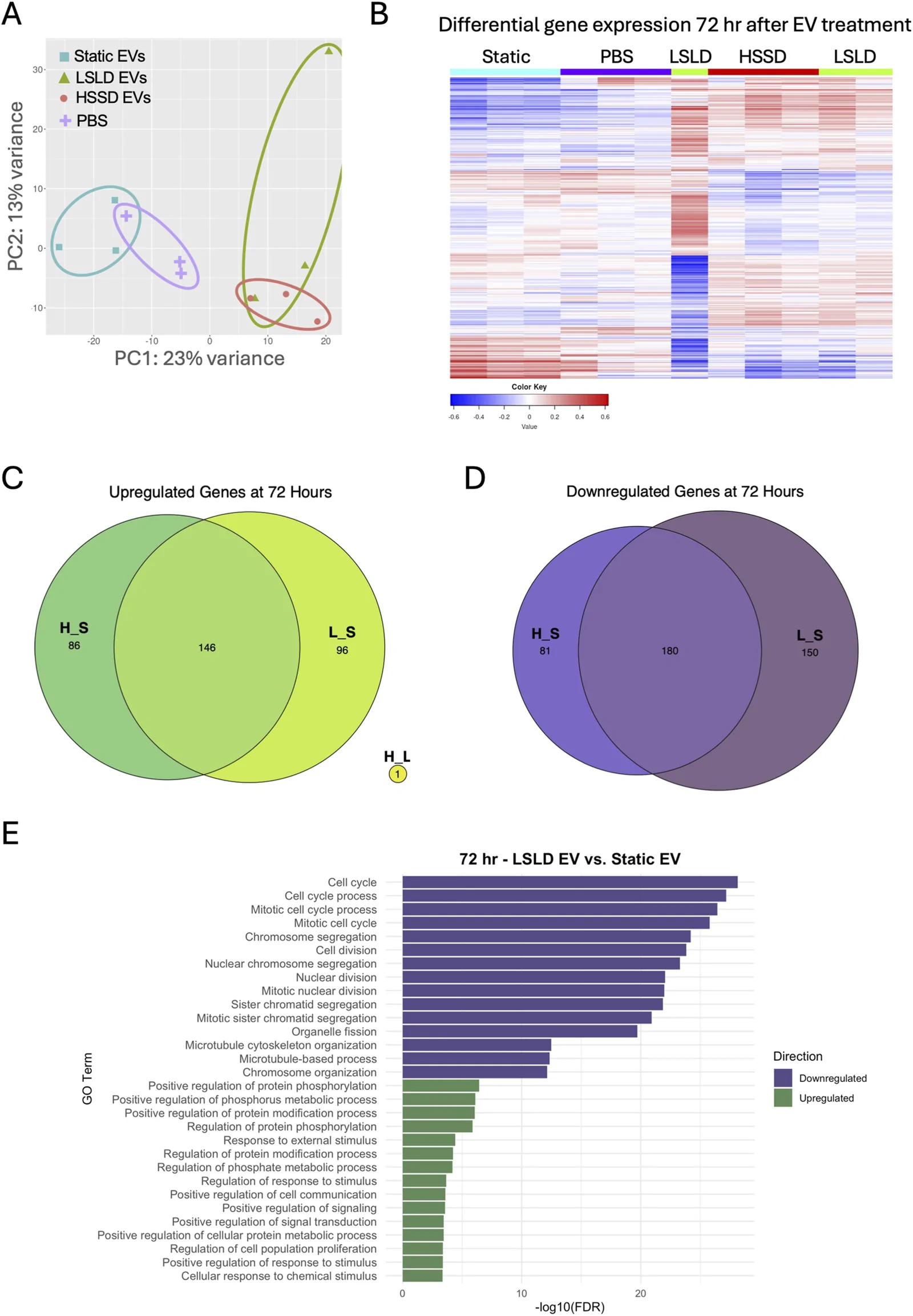
Mechanically strained myogenic cell-derived EVs induce distinct transcriptomic signatures in recipient myoblasts 72 h after EV exposure. **(A)** Principal component analysis (PCA) showing clustering of transcriptomic signatures of recipient myoblasts 72 h after treatment with EVs derived from Static, LSLD, or HSSD myoblasts, or PBS controls. **(B)** Heatmap showing the top 1,000 most variable genes of differentially expressed genes across treatment groups based on mRNA read counts. Red indicates relatively higher expression; blue indicates relatively lower expression. Blue bar = Static EV-treated; Purple bar = PBS treated; Red bar = HSSD EV-treated; Green bar = LSLD EV-treated **(C)** Venn diagram comparing upregulated genes in three pairwise comparisons: HSSD vs. Static EV-treated myoblasts (H_S), LSLD vs. Static EV-treated myoblasts (L_S), and HSSD vs. LSLD EV-treated myoblasts (H_L). **(D)** Venn diagram comparing downregulated genes in three pairwise comparisons: HSSD vs. Static EV-treated myoblasts (H_S), LSLD vs. Static EV-treated myoblasts (L_S), and HSSD vs. LSLD EV-treated myoblasts (H_L). **(E)** Gene ontology (GO) enrichment analysis of Biological Process terms for LSLD EV-treated myoblasts vs. Static EV-treated myoblasts 72 h after treatment. Purple shows downregulated GO terms and green shows upregulated GO terms. GO enrichment p-values were corrected for multiple hypothesis testing using the Benjamini–Hochberg false discovery rate (FDR) procedure, and significance is presented as -log_10_(FDR). GO terms with FDR <0.05 were considered statistically significant.

Recipient myogenic cells (n = 3 replicates per treatment group) were harvested for RNA sequencing 72-h after EV exposure to characterize the associated transcriptomic signatures and prolonged shifts in gene expression. PCA revealed that replicates predominantly clustered by treatment group (Figure 3A). A heatmap of the top 1,000 most variable genes demonstrated that myogenic cells treated with LSLD EVs or with HSSD EVs displayed similar gene expression patterns, while Static EV-treated and PBS vehicle-treated controls displayed more similar gene expression patterns to one another (Figure 3B).

Differential gene expression analysis was then performed between treatment groups. A total of 86 genes were uniquely upregulated in HSSD EV compared to Static EV-treated cells, while 146 genes were commonly upregulated in both HSSD EV vs. Static EV-treated cells and in LSLD EV vs. Static EV-treated cells, suggesting that EVs from both mechanical strain conditions induce a shared gene expression pattern that diverges distinctly from that of Static EV treatment. Ninety-six genes were uniquely upregulated in LSLD EV vs. Static EV-treated cells, indicating a gene expression pattern which is uniquely regulated by LSLD EV treatment. Only one gene was differentially upregulated with HSSD EV treatment compared to LSLD EV treatment, indicating that EVs from both strain conditions induce similar gene expression patterns 72 h post-treatment (Figure 3C).

A similar trend was observed in downregulated genes. Eighty-one genes were uniquely downregulated in HSSD EV compared to Static EV-treated cells, and 180 genes were commonly downregulated in both HSSD EV vs. Static EV-treated cells and in LSLD EV vs. Static EV-treated cells. One hundred and fifty genes were uniquely downregulated in LSLD EV vs. Static EV-treated cells. No genes were downregulated with HSSD EV treatment compared to LSLD EV treatment (Figure 3D).

Differentially expressed GO terms were then investigated between LSLD EV vs. Static EV-treated cells, as the most pronounced differences in gene expression 72 h following EV exposure were observed between these two treatment groups. The downregulated GO terms following treatment with LSLD EVs compared to Static EVs were largely related to cell cycle and cell division, suggesting that LSLD EVs are associated with a transcriptomic signature consistent with reduced proliferation 72 h after treatment (Figure 3E). The upregulated GO terms in myoblasts treated with LSLD EVs, including terms related to metabolism, response to an external stimulus, and signal transduction, correlate with a transcriptomic signature associated with myogenic maturation pathways. Overall, these transcriptomic results identify biological processes and gene targets that may be differentially regulated by EVs generated by cells subjected to mechanical strain.

### Predicted miR-222 and miR-148a targets are preferentially downregulated following treatment with LSLD-derived EVs

To assess whether transcriptomic changes in recipient cells were consistent with predicted targets of mechanically regulated EV-associated miRNAs, we tested whether predicted targets of miRNAs enriched in LSLD EVs were overrepresented among genes downregulated following LSLD EV treatment (Table 2).

**TABLE 2:** Fisher’s exact test results cross-validating predicted miRNA target gene lists against significantly downregulated mRNAs in recipient primary myogenic cells at 24 and 72 h post-LSLD EV treatment.

miRNA	Timepoint	Expected overlap	Observed overlap	Fold enrichment	p-value
*miR-148a-3p*	24 h	3.18	2	0.628	0.833
	72 h	12.0	19	1.58	0.0333*
*miR-222-3p*	24 h	2.30	5	2.17	0.0807
	72 h	8.63	17	1.97	0.00626**
*miR-222-5p*	24 h	1.96	5	2.55	0.0465*
	72 h	7.45	11	1.48	0.129

* = p-value <0.05, ** = p-value <0.01. miRNAs with FDR < 0.05 should be bolded.

**FIGURE 4:**
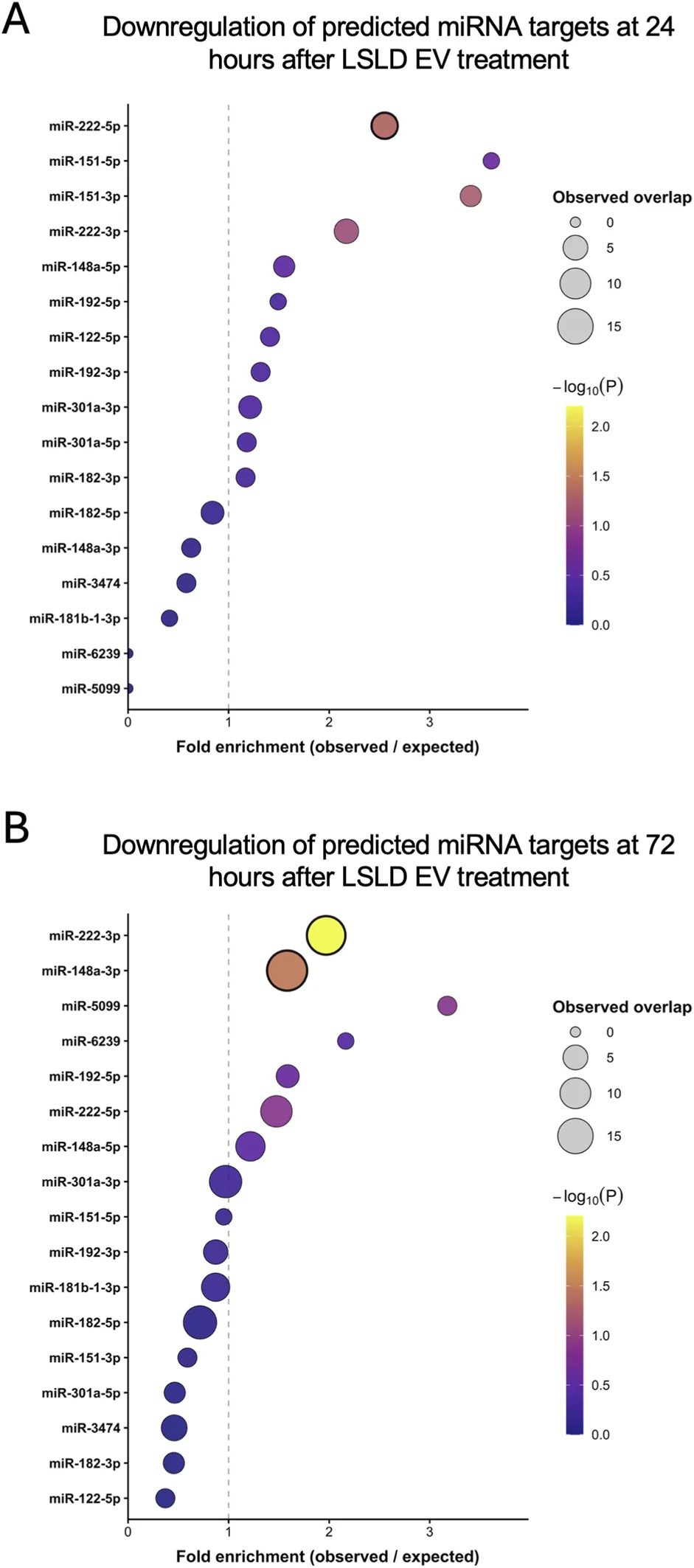
Enrichment of predicted miRNA target genes among downregulated genes following treatment with LSLD extracellular vesicles. **(A,B)** Bubble plots depict enrichment analyses performed on predicted targets of candidate miRNAs. MiRNAs were selected based on EV small RNA sequencing showing enrichment in LSLD EVs compared to Static EVs. Predicted target genes were evaluated for enrichment among genes downregulated following treatment with LSLD EVs relative to Static EVs at **(A)** 24 h and **(B)** 72 h. Enrichment analyses were performed using one-sided Fisher’s exact tests with the corresponding DESeq2 gene universe as the statistical background. The x-axis represents fold enrichment (observed overlap/expected overlap), bubble size indicates the number of overlapping genes between predicted miRNA targets and the downregulated gene set, and bubble color represents the one-sided Fisher’s exact p-value. The dashed vertical line indicates no enrichment (fold enrichment = 1).

This target cross-validation identified a significant overrepresentation of predicated miR-222-5p targets among downregulated genes in LSLD EV-treated cells at 24 h following treatment (p = 0.0465, Table 2; Figure 4A). These downregulated miR-222–5p predicted target genes included extracellular matrix and motility regulators (Adamtsl3, Cxadr, Itga3, Kctd12, Lrp2, Supplementary Table S1). Target cross-validation also identified a significant downregulation of miR-222–3p targets in LSLD EV-treated cells at 72 h following treatment (p-value = 0.00626, Table 2; Figure 4B). The 17 overlapping target genes represent key mitotic progressors (Mki67, Cdca3, Esco2, Exo1, Prr11) and cell cycle exit gatekeepers (Esr1 and Cdkn1c/p57) (Supplementary Table S1). Predicted target sets corresponding to the two mature miR-222 arms showed different temporal enrichment patterns, with miR-222–5p predicted targets enriched among downregulated genes at 24 h and miR-222–3p predicted targets enriched at 72 h (Table 2). Because mature miRNA arms were not quantified in the EV samples, these findings should not be interpreted as evidence of time-dependent enrichment or activity of the individual miR-222 arms.

Notably, miR-148a-3p also demonstrated a highly significant overrepresentation of predicted targets among genes downregulated in recipient cells 72 h following LSLD EV treatment (Table 2; Figure 4B). Intersecting the predicted targets of miR-148a-3p with the downregulated transcripts demonstrated a significant overlap of 19 genes (p-value = 0.0333). These 19 downregulated target genes include regulators of mitotic division and spindle progression (Aurkb, Ccnf, E2f7, Kif14, and Garem1), regulators of satellite cell self-renewal (Esr1), and regulators of extracellular matrix remodeling (Fbn1, Frem, Itga4, Syne2, Adamts15, Parm1) (Supplementary Table S1).

To determine whether HSSD EVs induced a distinct regulatory effect on the downstream targets of similar miRNAs, a parallel targeted enrichment analysis was performed on genes downregulated following HSSD EV treatment vs. Static EV treatment. Interestingly, at 24 h post-treatment, HSSD EV exposure resulted in a significant overrepresentation of downregulated target genes for miR-192–3p (p-value = 0.03893), miR-182–3p (p-value = 0.001956) and miR-182–5p (p-value = 0.008523) (Supplementary Figure S6A; Supplementary Table S2). By 72 h post-treatment, HSSD EV exposure resulted in a significant overrepresentation of downregulated target genes for miR-222–3p (p-value = 0.04401) and miR-222–5p (p-value = 0.03682) (Supplementary Figure S6B; Supplementary Table S2).

The enrichment of predicted miR-222 target sets among downregulated genes observed at 72 h is comparable between the LSLD and HSSD EV groups, underscoring a shared transcriptomic trajectory induced by both mechanical strain regimens. However, the temporal pattern of predicted miR-222 target-set enrichment differed between treatment groups: enrichment of the miR-222–5p predicted target set at 24 h was observed only following LSLD EV treatment. Furthermore, in contrast to the shared miR-222 signature at 72 h following treatment with either LSLD EVs or HSSD EVs, the significant downregulation of miR-148a-3p targets driven by LSLD EVs at 72 h was completely absent in the HSSD group. This difference in enrichment of the predicted miR-148a-3p target set is consistent with distinct, protocol-specific transcriptomic signatures following LSLD and HSSD EV treatment, though it does not establish miR-148a-3p-specific regulation.

### Mechanical strain of EV-producing myogenic cells reduces proliferation and enhances differentiation properties of myogenic cell-derived EVs

Because EVs derived from strained myogenic cells were predicted to downregulate GO terms related to cell cycle and cell proliferation relative to Static EVs, we next aimed to determine how EVs isolated from myoblasts under each strain condition influence proliferation of recipient myoblasts *in vitro*. DNA synthesis was therefore monitored over time following treatment with EVs derived from Static, HSSD, or LSLD strain conditions, alongside PBS vehicle controls.

**FIGURE 5:**
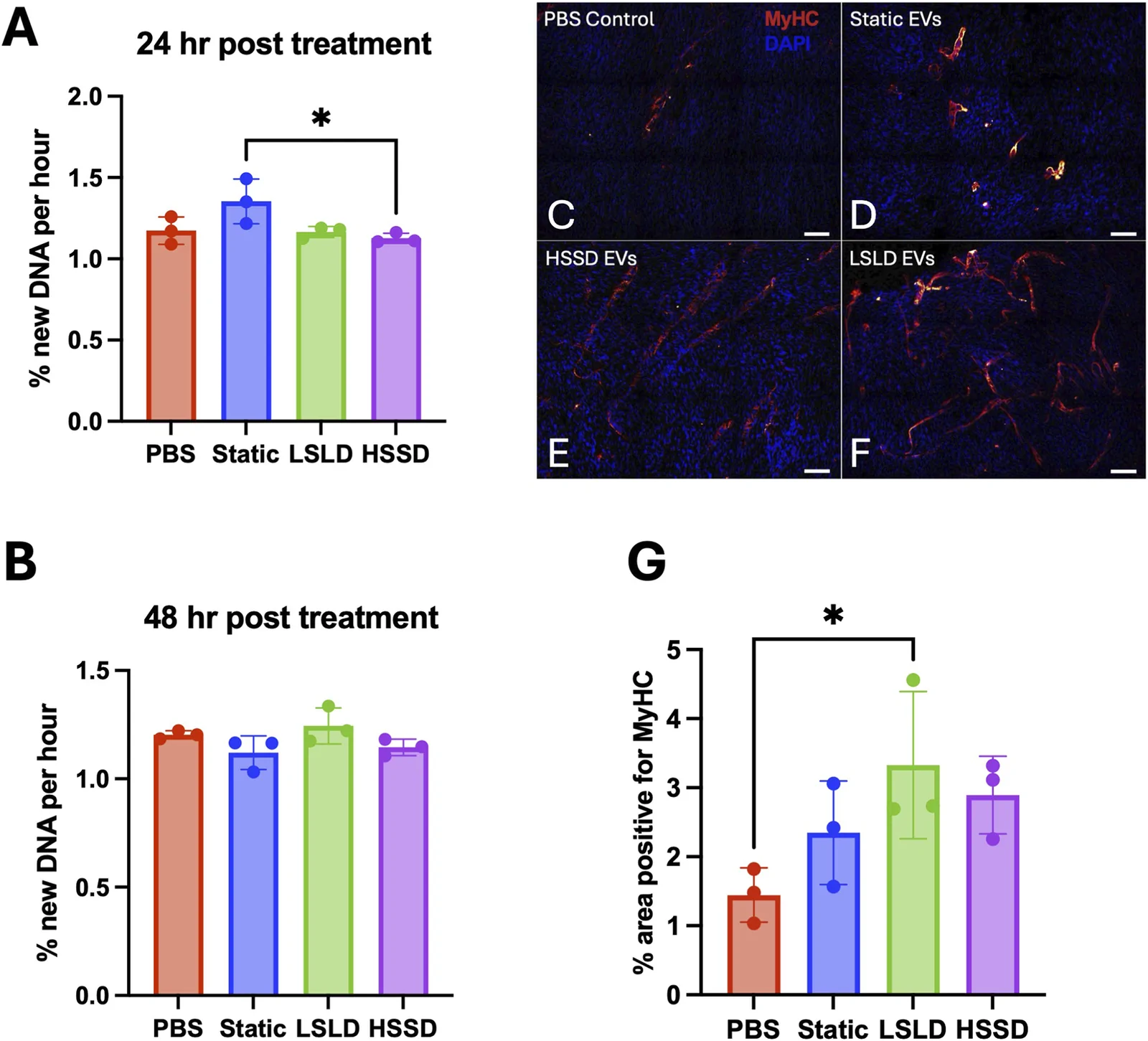
EVs derived from mechanically strained myogenic cells increase myotube differentiation. **(A,B)** Quantification of DNA synthesis by measuring the percentage of new DNA synthesized per hour using D_2_O labeling at **(A)** 24 h and **(B)** 48 h post-treatment. Statistical analyses were performed using one-way ANOVA with Tukey’s multiple comparisons test to compare all experimental groups (LSLD EV, HSSD EV, Static EV, and PBS vehicle control) to one another. * Indicates p < 0.05. Only pairwise comparisons that reached statistical significance (p < 0.05) are indicated. **(C–F)** Representative images of primary myocytes stained for myosin heavy chain (MyHC, red) and DAPI (blue) following 2 days of treatment with **(C)** PBS **(D)** Static EVs **(E)** HSSD EVs, or **(F)** LSLD EVs derived from primary myoblasts. Scale bars represent 100 μm **(G)** Within each replicate well, eight randomized areas were imaged in a 3 × 3 grid pattern. ImageJ was used to quantify the percent area positive for MyHC. Statistical analyses were performed using one-way ANOVA with Tukey’s multiple comparisons test to compare the average MyHC-positive area across each of the three experimental replicates. * Indicates p < 0.05; ns indicates no statistical significance (p > 0.050).

To directly measure DNA synthesis, deuterium oxide (D_2_O) incorporation assays were performed at 24- and 48-h post-treatment. At 24 h post-EV treatment, Static EV-treated myogenic cells had the highest rate of DNA synthesis. The rate of DNA synthesis of Static-EV-treated cells was significantly higher than HSSD EV-treated cells (p = 0.0427), and showed a non-significant increase compared to all other treatment groups (Figure 5A). No significant differences in rate of DNA synthesis were identified between any treatment groups at 48 h post-treatment (Figure 5B). Together, these data indicate that mechanical strain transiently attenuates the proliferative effects of myogenic cell-derived EVs. While this reduction was modest and only apparent at 24 h post-treatment, it indicates that mechanical strain may modulate EV function away from promoting proliferation, potentially favoring later myogenic processes such as differentiation. Cell confluence of primary myogenic cells was then monitored for 4 days following EV exposure. No significant differences were observed in cell confluence between any treatment groups (Supplementary Figure S7), indicating that myogenic EVs do not affect primary myogenic cell proliferation to a level that is appreciable in observable cell confluence.

Finally, we sought to determine the impact of EVs derived from mechanically strained myogenic cells on myogenic differentiation. Following a 48-h treatment of myogenic cells with EVs from each strain condition, cells were fixed and immunostained for MyHC, a marker of mature myotubes. Representative images of cells from each treatment group depict MyHC expression in HSSD EV-, LSLD EV-, Static EV-, and PBS vehicle-treated groups (Figures 5C–F).

Quantification of the percent area positive for MyHC demonstrated a statistically significant increase in MyHC in LSLD EV-treated cells compared to the PBS vehicle control (p = 0.0345, Figure 5G). However, direct pairwise comparisons of LSLD EV vs. Static EV and HSSD EV vs. Static EV represented an upward trend that did not reach statistical significance (p > 0.05). Overall, these results indicate that the LSLD mechanical strain protocol is associated with an increase in the myogenic marker MyHC in recipient myoblasts 48 h after treatment compared to PBS controls.

## Discussion

This study investigated how mechanical strain influences the molecular cargo and biological function of EVs secreted by primary murine myogenic cells. Our findings demonstrate an association between the application of biophysical strain, subsequent alterations in the miRNA cargo of EVs, and a functional shift in EV bioactivity toward a differentiation-associated phenotype. Specifically, mechanical strain was associated with a distinct EV miRNA profile, including the selective enrichment of miR-222 and miR-148a in EVs derived from the LSLD protocol. When applied to naïve recipient myoblasts, EVs isolated from strained cells induced protocol-specific transcriptional signatures associated with the suppression of proliferation pathways and the promotion of differentiation pathways. These transcriptomic findings are functionally supported by a reduction in early active DNA synthesis following HSSD EV treatment, and an increase in terminal MyHC expression following LSLD EV treatment. Our miRNA target cross-validation analysis demonstrated that the predicted targets of miR-222, which is selectively enriched in LSLD EVs compared to HSSD EVs and to Static EVs, are highly overrepresented among downregulated transcripts in recipient cells at 24 and 72 h following LSLD EV treatment. Together, these data demonstrate that mechanical strain is a potent stimulus for shifting the molecular cargo of EVs and guiding recipient myogenic cell behavior.

The two mechanical strain regimens utilized in this study included 1) the LSLD regimen, which subjected cells to a low-magnitude, continuous load, and 2) the HSSD regimen, which subjected cells to cycles of brief, high-intensity loading. Both strain conditions resulted in the common enrichment of broadly responsive miRNAs, including miR-301 and miR-6239. Because miR-301 and miR-6239 are commonly enriched in both strain groups, they likely reflect a conserved, baseline cellular response to mechanical strain rather than the drivers of the specific phenotypic differences observed between LSLD EV and HSSD EV treatments.

Consistent with previous studies (Mullen et al., 2023), we found that EV amount is influenced by mechanical strain, with the HSSD regimen resulting in a 2.5-fold increase in primary myogenic EV production. Furthermore, our study found that the muscle-specific miRNA, miR-206, was the most abundant transcript packaged within our EVs across all experimental conditions. This high miR-206 baseline enrichment aligns with previous literature demonstrating that muscle cells release EVs enriched with miR-206 during hypertrophy, injury recovery, and muscle niche remodeling (Fry et al., 2017; Watanabe et al., 2022; Yu et al., 2024). While miR-206 abundance remained stable regardless of the specific strain regimen applied in the current study, its significant abundance confirms the highly myogenic origin of our isolated EV pool.

We found that the effects of EVs derived from strained myogenic cells were most pronounced by 72 hours-post-treatment. LSLD EVs, in particular, suppressed cell cycle-related genes and upregulated genes related to cell metabolism. The upregulation of metabolic genes may reflect increased energy demands associated with terminal differentiation and maturation of myotubes, as mature myotubes rely on efficient metabolic activity, particularly oxidative phosphorylation, to support contractile protein synthesis (Ryall, 2013). The downregulation of cell cycle-related genes observed at 72 h further corresponds with the reduction in DNA synthesis at 24 h and the subsequent increase in MyHC-positive area, providing supporting evidence that EVs derived from mechanically strained myogenic cells may favor a post-mitotic, pro-myogenic state.

We identified a highly significant overrepresentation of predicted targets for both miR-222–3p and miR-148a-3p among the genes downregulated in LSLD EV-treated recipient cells 72 h after EV treatment. The enrichment of predicted miRNA targets among downregulated cell-cycle-related genes provides a hypothesis-generating association that may warrant future mechanistic investigation. The predicted targets of the two miR-222 mature arms showed different temporal patterns following LSLD EV treatment. Predicted miR-222–5p targets were enriched among downregulated genes at 24 h, whereas predicted miR-222–3p targets were enriched at 72 h. Because our small RNA sequencing quantified the miR-222 precursor rather than the individual mature 3p and 5p arms, we cannot determine which mature species was enriched within the EVs. One possible explanation is that the transcriptome of recipient myogenic cells changes over time, correlating with distinct predicted target profiles in recipient cells at 24 versus 72 h. It is also possible that the later enrichment of miR-222–3p targets reflects potential indirect downstream transcriptomic cascades, following early EV exposure. Future studies using mature arm-specific quantification and functional validation will be needed to determine whether both miR-222 arms are transferred by EVs.

In contrast to the transcriptomic changes observed at 72 h with LSLD EV treatment, HSSD EVs induced a more rapid and transient transcriptomic response. At 24 h post-treatment, recipient myogenic cells exposed to HSSD EVs displayed a significant downregulation of miR-192–3p and miR-182–3p/5p targets. These suppressed targets included extracellular matrix and cell motility regulators (including *Thbs1*, *Fn1*, and *Timp3*). Interestingly, these specific miRNAs were not relatively enriched in the HSSD EV cargo compared to Static EVs. Similarly, at 72 h after HSSD EV treatment, predicted targets of miR-222–3p/-5p were significantly downregulated in recipient myogenic cells, despite miR-222 not being relatively enriched in HSSD EV cargo. It is possible that abundant baseline miRNAs (such as miR-206 and miR-128–1) may work cooperatively with strain-enriched species (such as miR-301 and miR-6239) to co-repress shared genes, leading to synergistic target silencing (Chen et al., 2017). While HSSD EVs only showed miR-222 target enrichment at 72 h after treatment, LSLD EVs drove a significant early downregulation of miR-222–5p targets at 24 h after treatment.

The current study establishes association, not causation, between specific EV miRNAs, transcriptomic changes, and observed functional outcomes. To establish a direct causal link between miRNA changes within EVs derived from strained myogenic cells and functional outcomes, future studies utilizing loss-of-function experiments (i.e., antagomirs to inhibit candidate mature sequences associated with the miR-222 and miR-148a loci) and gain-of-function experiments (i.e., loading EVs from static cells with specific miRNA mimics) are critical next steps to fully characterize the biologic mechanisms driving these results.

Interestingly, LSLD and HSSD EVs showed only transiently reduced proliferation compared to Static EVs. The apparent reduction in DNA synthesis at 24 h post-treatment following HSSD EV treatment, without subsequent differences in cell confluence at later timepoints, suggests that the early proliferative effects of EVs may be subtle, or compensated for by other regulatory mechanisms in myogenic cells cultures. For example, differences in proliferation rates may be obscured by other regulators of cell confluence including contact inhibition and other growth-limiting factors. Future research is therefore required to determine whether these transient proliferative shifts represent a functional commitment to terminal differentiation.

While the current study investigated the effects of primary myogenic cell-derived EVs on naïve myogenic cells, it would be valuable for future studies to investigate the impact of mechanically strained EVs on other cell types involved in muscle regeneration. Given that other studies have demonstrated a role for muscle EVs in regulating ECM deposition by fibroblasts (Fry et al., 2017) and adipogenic differentiation of fibroadipogenic progenitors (Yu et al., 2024), it would be of interest to investigate how mechanical strain impacts these particular functions of myogenic EVs on recipient fibroblasts. Our miRNA sequencing data identified a broad set of GO terms including neurogenic processes, angiogenesis, and osteogenic pathways, suggesting that these EVs may have broader targets beyond myoblasts.

This study has several limitations which should be noted. First, the reliance on primary myogenic cells isolated from a single biological donor mouse (a 13-week-old female C57BL/6 mouse). While using a single donor limits the immediate generalizability of our findings, this controlled approach was chosen to eliminate genetic and inter-animal variability. This allowed us to strictly isolate the effects of mechanical strain on EV production, cargo, and bioactivity from a consistent cell source. However, to ensure these findings are not unique to an individual animal, future studies must validate the observed shifts in EV miRNA signatures and myogenic differentiation capacity across multiple independent primary donors. Second, while our rigorous serial pre-plating protocol significantly enriched the primary myogenic population (evidenced by morphological homogeneity, mature MyHC expression, spontaneous contractile activity, and muscle-specific miRNA EV cargo), we cannot completely exclude the minor contribution of non-muscle interstitial cells, such as fibroblasts, to our donor EV pool. Future studies utilizing fluorescence-activated cell sorting (FACS) to isolate highly pure myogenic progenitors prior to EV collection will be valuable to fully exclude fibroblasts from the EV donor pool.

Third, to capture sensitive, baseline transcriptional changes before culture-induced drift occurred, we utilized early-passage primary cells (P6) for recipient RNA-sequencing. However, the subsequent functional assays (DNA synthesis and MyHC immunofluorescence) were performed using passage 14 (P14) primary cells. This was necessary due to the high quantity of primary cells required to for phenotypic assays. While our P14 cells successfully retained their capacity to exit the cell cycle and undergo terminal differentiation, we acknowledge that repeated passaging may have altered their baseline responsiveness compared to P6 cells, which must be considered when comparing the transcriptomic and phenotypic data. Future work should therefore validate these biological endpoints using passage-matched cultures where feasible.

Fourth, the LSLD and HSSD protocols differed not only in strain magnitude, but also in duration of rest intervals. Future work is necessary to isolate the specific effects of each strain parameter (i.e., strain magnitude vs. rest interval) on EV cargo and function. Fifth, our assessment of myogenic differentiation relied on MyHC expression, and while this is a key terminal skeletal muscle differentiation marker, future studies would benefit from additional differentiation metrics such as fusion index, myogenic regulatory factor expression, and Pax7 expression. Additional protein-level validation via Western blot or qPCR also represents a critical avenue for future investigations building on this work to validate the impact of key miRNAs identified in our study on predicted target genes.

Sixth, our analysis focused exclusively on miRNA cargo, leaving the protein, lipid, and other long RNA cargo within these EVs unassessed. Because these non-miRNA components likely vary between the LSLD and HSSD conditions, characterizing the EV proteome is a critical next step to identifying what drives functional differences between LSLD EVs and HSSD EVs. Finally, it remains unclear if the results described in this study are due to qualitative differences in EV cargo, or to differences in quantitative cargo load per EV. Our study normalized EV treatments by particle number and did not measure quantitative total cargo within EVs following strain. Future work is necessary to evaluate changes in cargo load per EV following mechanical strain, and to determine whether changes in cargo load may contribute to functional outcomes.

In summary, this study provides evidence that mechanical strain modulates EV miRNA cargo and is associated with a differentiation-related phenotype in EVs derived from primary myogenic cells. While highly abundant baseline regulators such as miR-206 remain stable components of myogenic EVs, specific mechanical strain regimens, particularly LSLD, selectively enrich EVs with unique miRNAs, including miR-222 and miR-148a. These strain-associated miRNAs and their predicted target signatures provide candidates for future studies examining the molecular basis of recipient-cell responses. Ultimately, this study establishes a biophysical strategy to tune and optimize EV cargo to guide recipient cell behavior without the need for genetic or pharmacological manipulation. These findings lay a foundational framework to evaluate the efficacy of strain-optimized EVs in physiologically relevant models, such as injured or sarcopenic skeletal muscle, advancing EV potential for targeted regenerative medicine applications.

## Data Availability

The mRNA-sequencing and miRNA-sequencing datasets generated in this study have been deposited in the NCBI Gene Expression Omnibus (GEO) and are publicly available under accession numbers GSE343064 and GSE342746, respectively.
