# Supplementary material for: Biomechanical strain alters the microRNA cargo of primary myogenic cell-derived extracellular vesicles associated with a differentiation phenotype
Source: Front Cell Dev Biol. 2026 Sep 10;14:1858881. doi: 10.3389/fcell.2026.1858881 (PMC13601279; doi:10.3389/fcell.2026.1858881)
Supplement: Supplementary file 1 [file DataSheet3.pdf]

Supplementary Material

Supplementary Figures

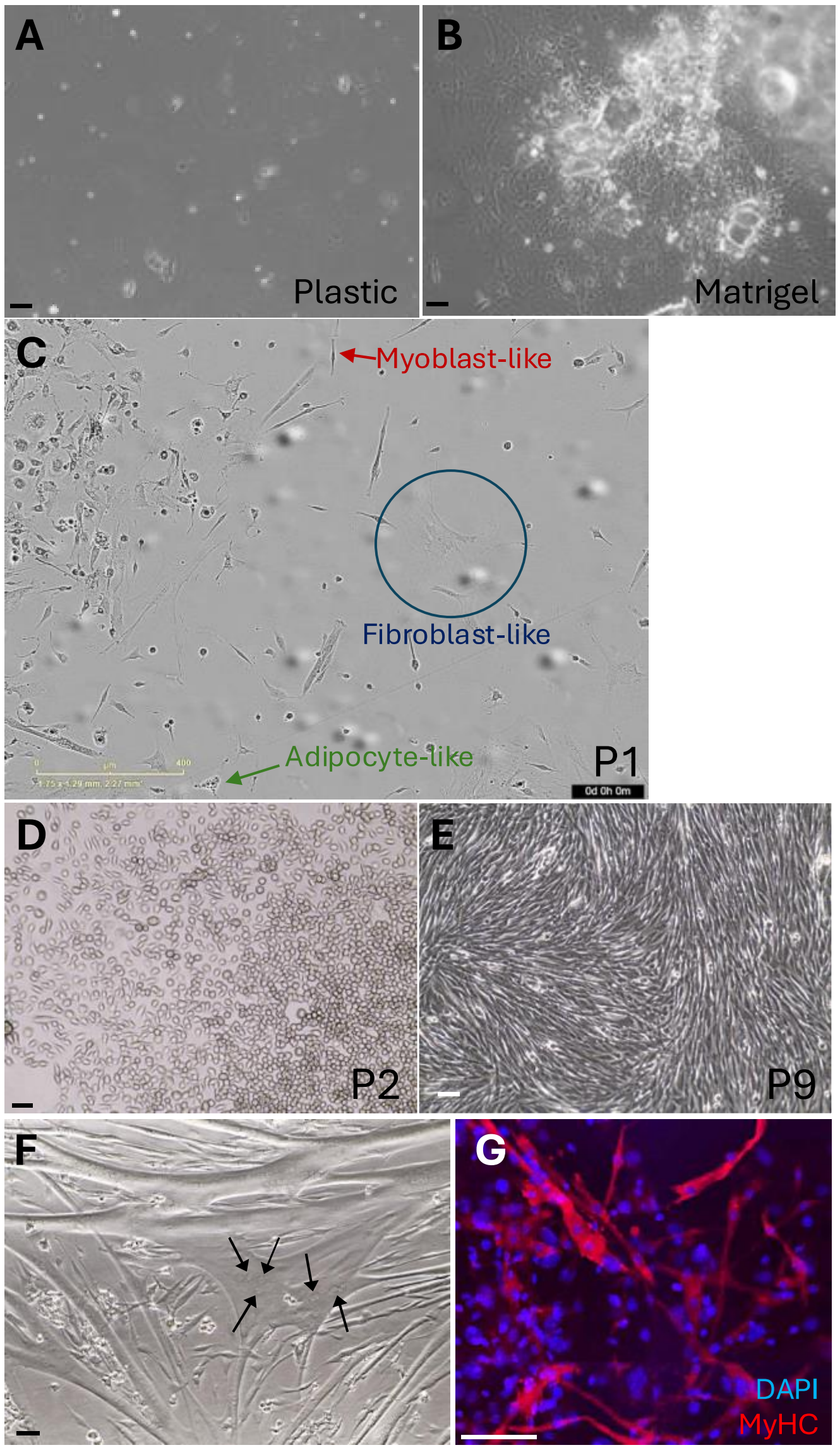

Supplemental Figure 1. Expansion of cells from skeletal muscle tissue harvest. A-B) Comparison of cell growth 2 days after plated minced muscle tissue on A) a plastic tissue-culture treated flask and B) a Matrigel-coated tissue-culture treated flask. Scale bar is 100 µm. C) Adherent cells at passage 1, following one round of pre-plating to deplete fibroblasts from culture. Three distinct cell types can be appreciated: 1) large, stellate-shaped cells with a branched cytoplasm and prominent nucleoli resembling fibroblasts (blue circle), 2) small cells with multiple clear, refractive vacuoles characteristic lipid droplets, resembling adipocytes (green arrow), and 3) small, spindle shaped cells resembling myoblasts (red arrow). Scale bar is 400 µm. D) Brightfield image of a homogeneous population of small, round cells resembling

undifferentiated satellite cells at passage 2, after two rounds of pre-plating to deplete fibroblasts. E) Brightfield image of a homogenous population of spindle-shaped cells resembling myoblasts at passage 9. F) Brightfield image of myocytes after differentiation of myoblasts into multinucleated myotubes. Arrows point to several nuclei within a large myotube. G) Immunofluorescence imaging of differentiated primary myotubes stained for MyHC (red) and DAPI (blue). Scale bar is 100 µM in D-G.

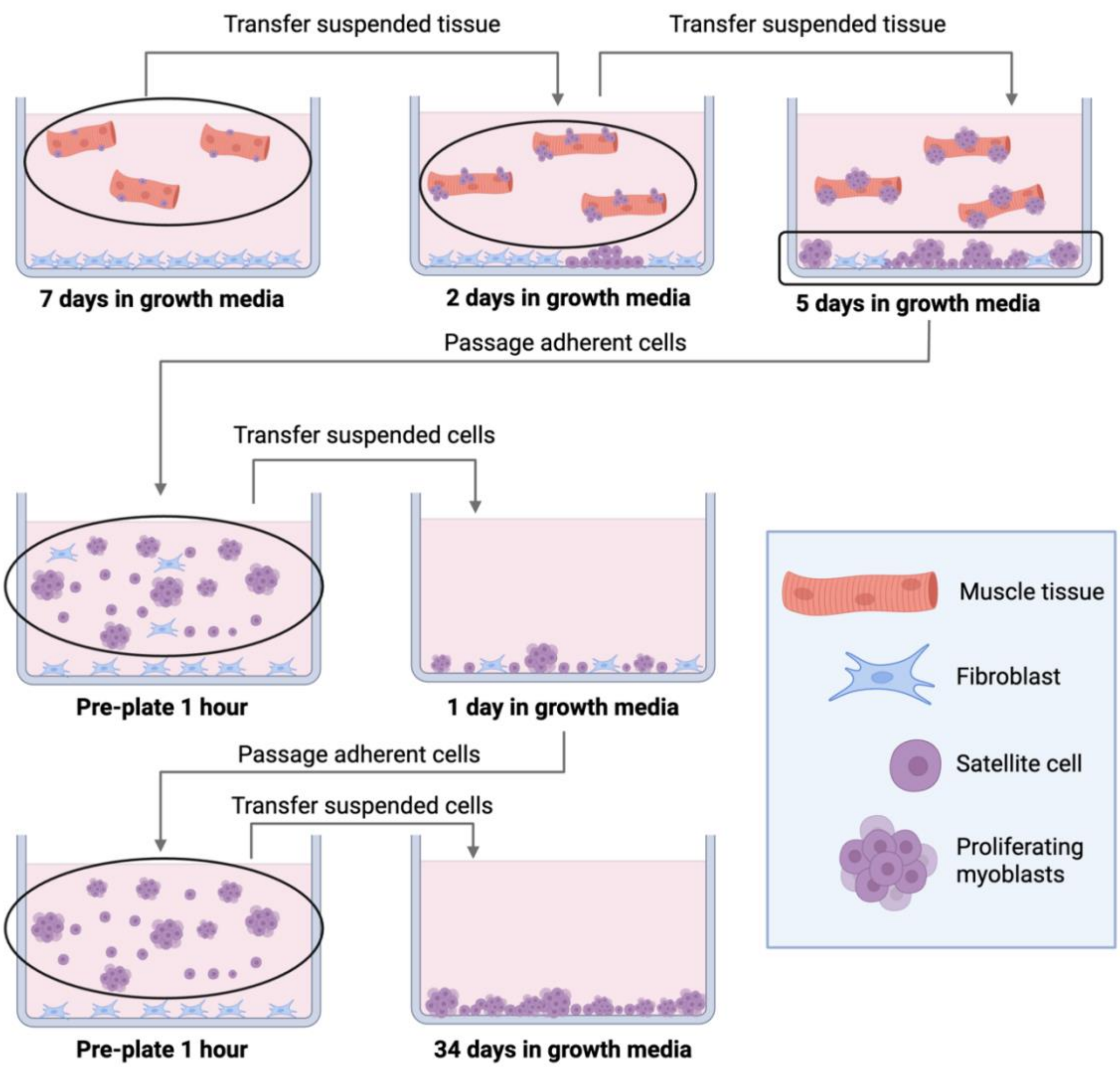

Supplemental Figure 2. Schematic demonstrating protocol for isolation of primary myogenic cells. Hindlimb muscles were harvested from a female 13-week-old C57BL/6 mouse, and myoblasts were cultured on Matrigel-coated flasks as shown.

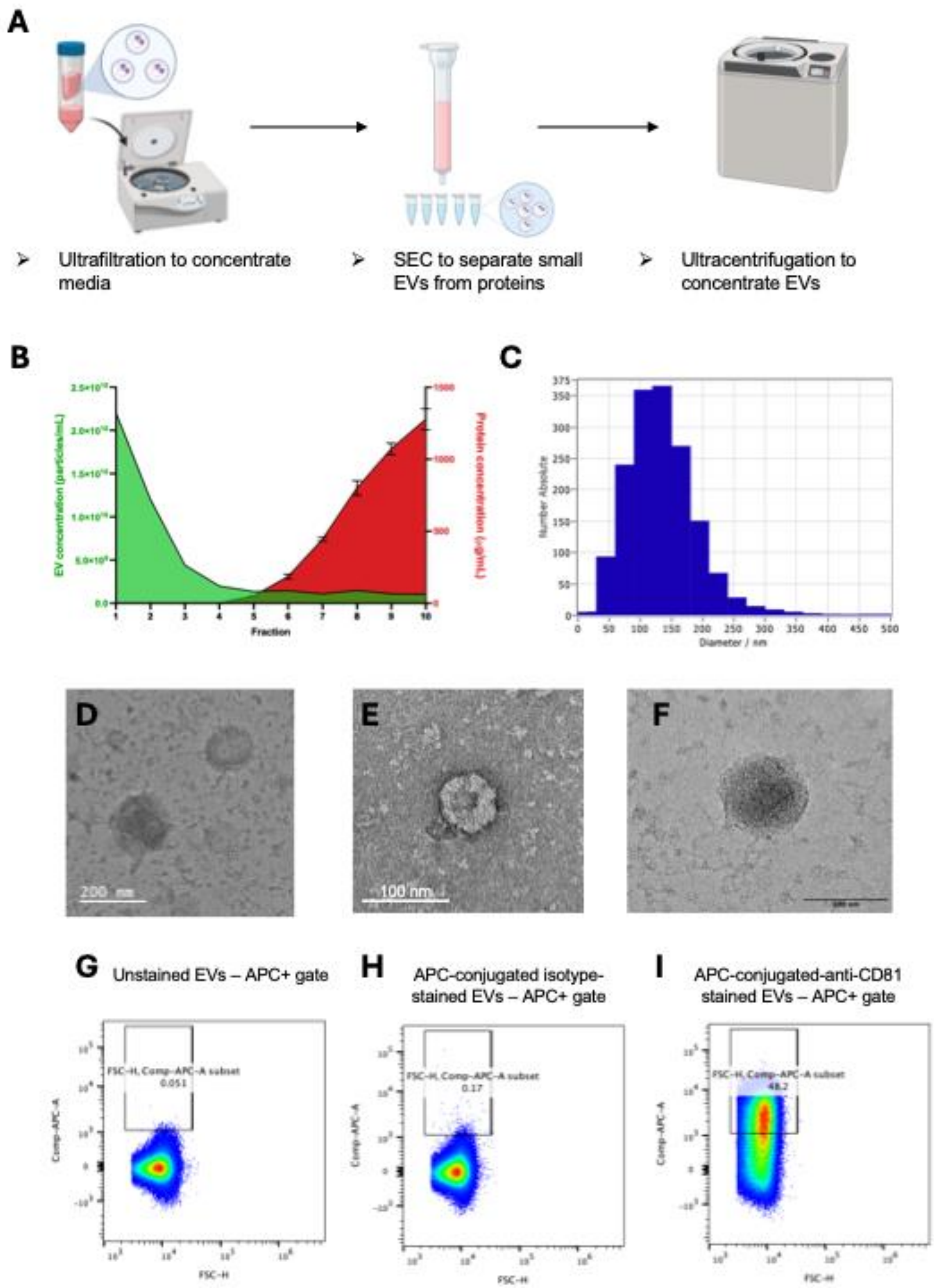

Supplementary Figure 3. Primary C57BL6 myogenic cells produce CD81+ EVs. (A) Schematic of methods used to isolate EVs including ultrafiltration, size exclusion

chromatography (SEC), and ultracentrifugation. (B) EV and protein concentration within 10 fractions isolated after SEC was performed on 25 mL of conditioned myoblast cell culture media. EV concentration was determined using nanoparticle tracking analysis (NTA) and protein concentration was determined using a BCA assay. (C) Representative size distribution and concentration plot of EVs within fraction 1, which had the highest concentration of EVs, determined using NTA. (D) Low-magnification scanning transmission electron microscopy (STEM) image showing two round EVs. Scale bar is 200 µm. (E) Higher-magnification STEM image depicting a single EV with characteristic round morphology. Scale bar is 100 nm. (F) TEM image (reverse contrast) of a single EV, displaying a round morphology and approximate 100 nm diameter. Scale bar is 100 nm. (G-I) Flow cytometry of primary myoblast EVs demonstrating (G) unstained EVs, (H) EVs incubated with APC-conjugated isotype control antibody, and (I) EVs incubated with anti-CD81-APC antibody. Rectangle shows APC+ gate, and the numeric value represents the percentage of particles within the gate.

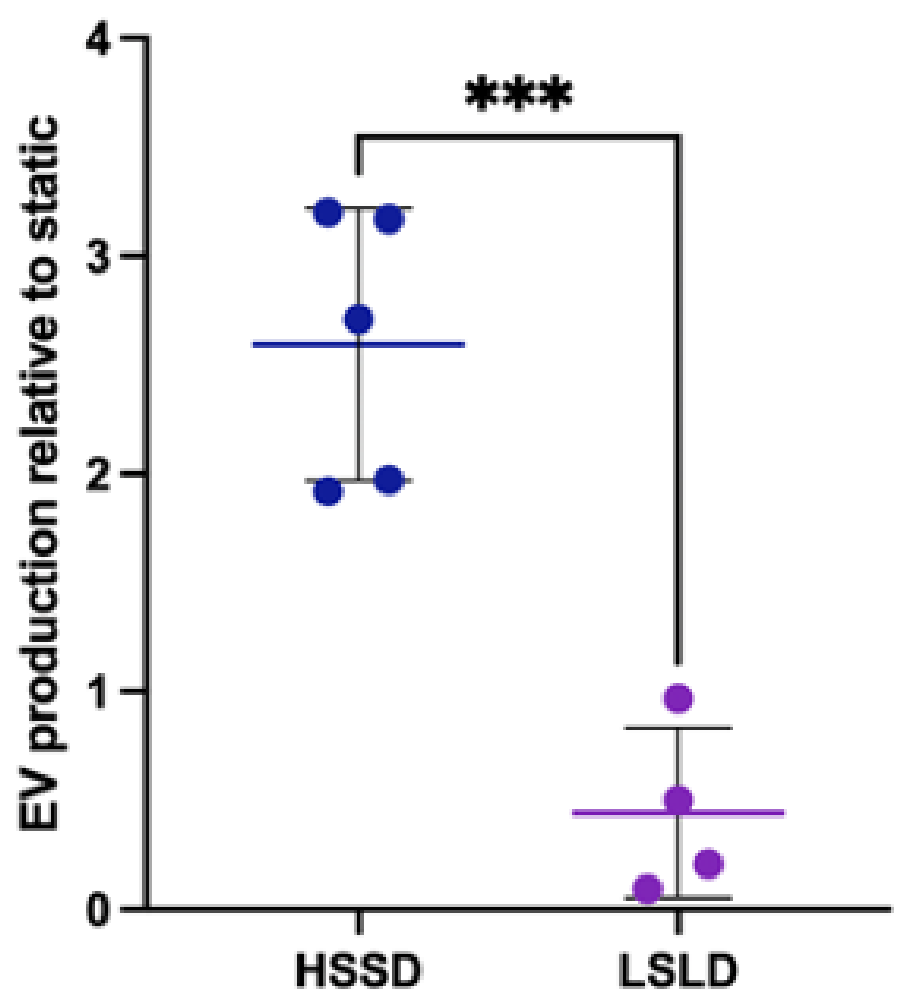

Supplemental Figure 4. Total EV production by primary myogenic cells is modulated by mechanical strain. The HSSD regimen resulted in approximately 2.5 times more EV production relative to Static. The LSLD regimen resulted in approximately half the amount of EV numbers compared to Static. Static = unstrained cells, HSSD = High Strain Short Duration, LSLD = Low Strain Long Duration. Statistical comparisons were performed using a t-test. *** indicates $p < 0.001$.

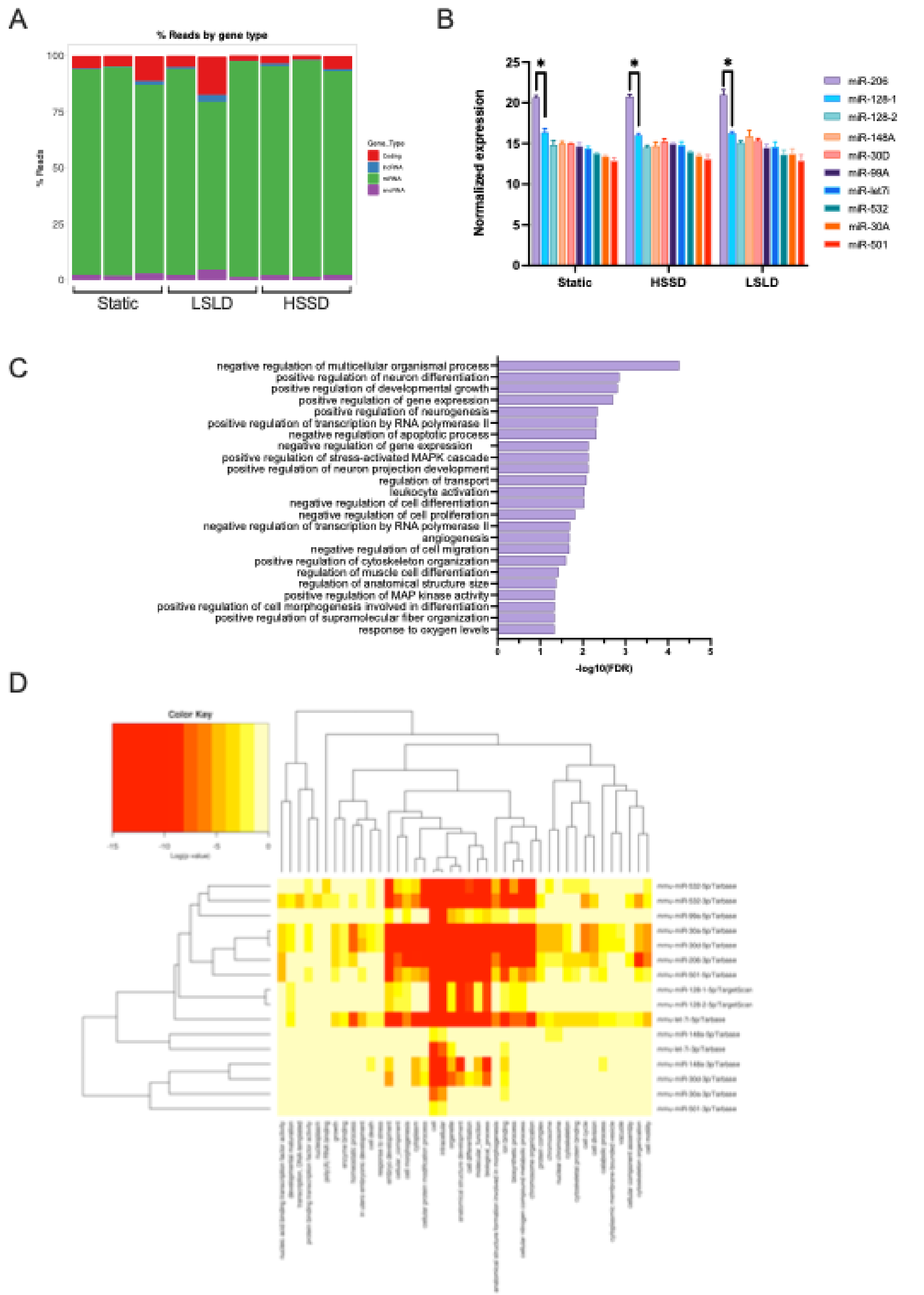

Supplemental Figure 5: Small RNA-sequencing of EVs produced by differentiating myogenic cells following mechanical strain. (A) Percentage of total reads demonstrates that the majority of small RNAs within myoblasts EVs are microRNAs (miRNAs). (B) Normalized expression values of top 10 most abundant miRNAs for each strain condition (mean normalized expression values for n=3 EV samples per condition, error bars represent standard deviation).

Unpaired t-tests were used to statistically compare normalized count data between miR-206 and miR128-1 (the two most abundant miRNAs in each sample); $p < 0.001$. (C) Terminal nodes of enriched gene ontology (GO) terms of the predicted mRNA targets of miR-206, which was the most abundant miRNA in all samples. (D) Heatmap depicting shared enriched GO biological process terms of the top 10 most abundant miRNAs in all EV samples.

A

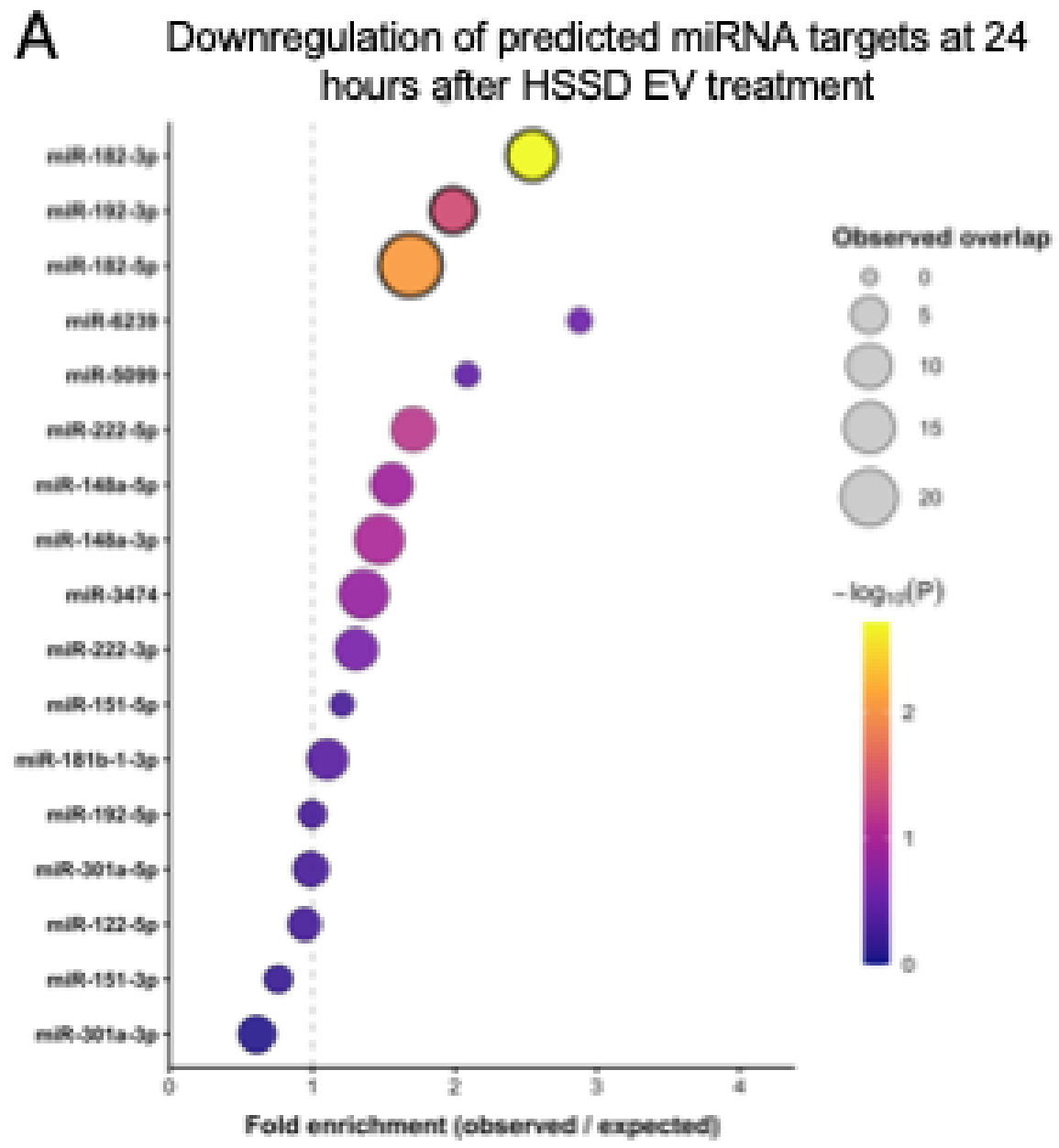

B

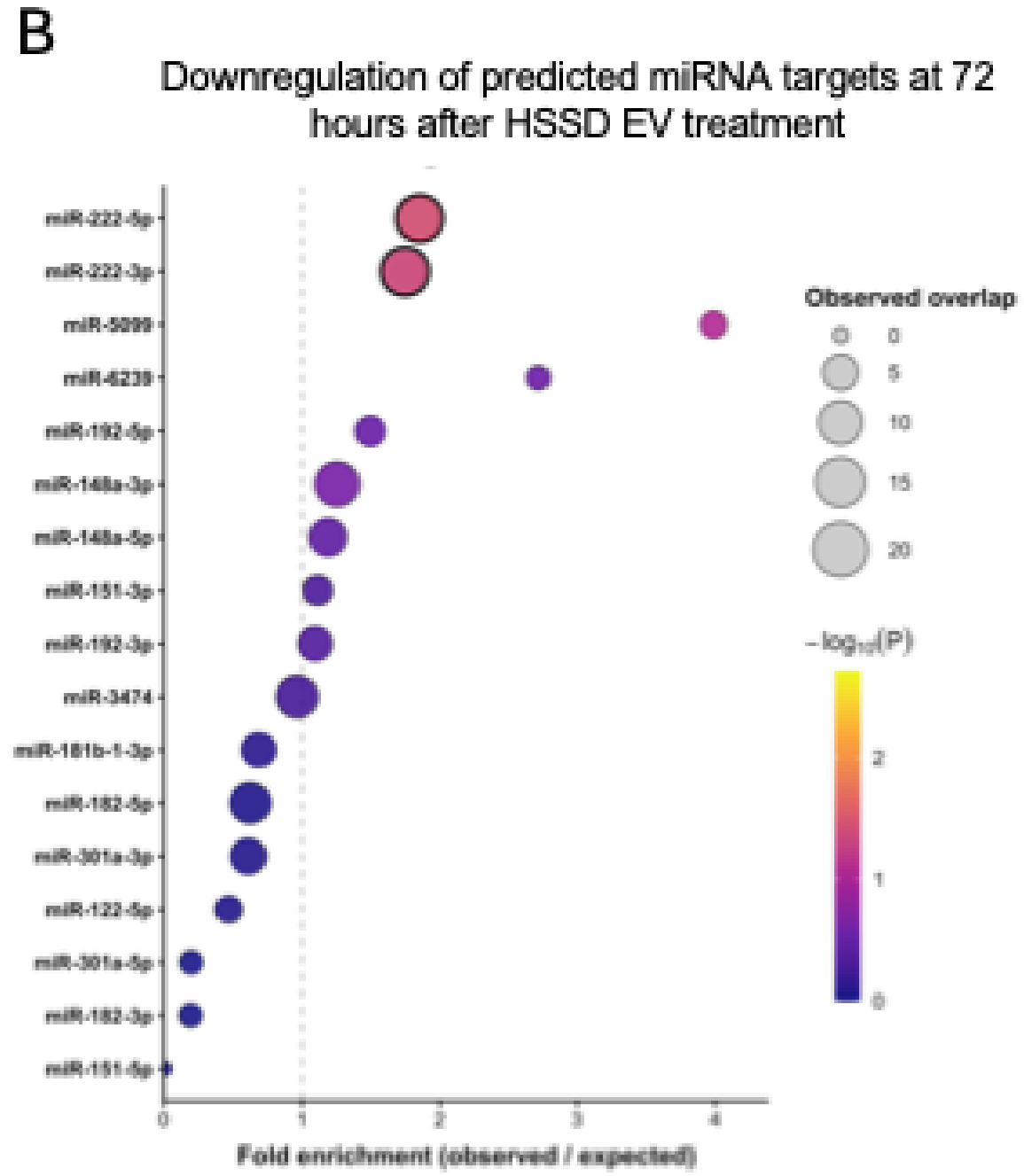

Supplemental Figure 6: Enrichment of predicted miRNA target genes among downregulated genes following treatment with HSSD extracellular vesicles. (A-B) Bubble plots depict enrichment analyses performed on predicted targets of candidate miRNAs. MiRNAs were selected based on EV small RNA sequencing showing enrichment in LSLD EVs compared to Static EVs. Predicted target genes were evaluated for enrichment among genes downregulated following treatment with HSSD EVs relative to Static EVs at (A) 24 hours and (B) 72 hours. Enrichment analyses were performed using one-sided Fisher's exact tests with the corresponding DESeq2 gene universe as the statistical background. The x-axis represents fold enrichment (observed overlap/expected overlap), bubble size indicates the number of overlapping genes between predicted miRNA targets and the downregulated gene set, and bubble color represents the one-sided Fisher's exact p-value. The dashed vertical line indicates no enrichment (fold enrichment = 1).

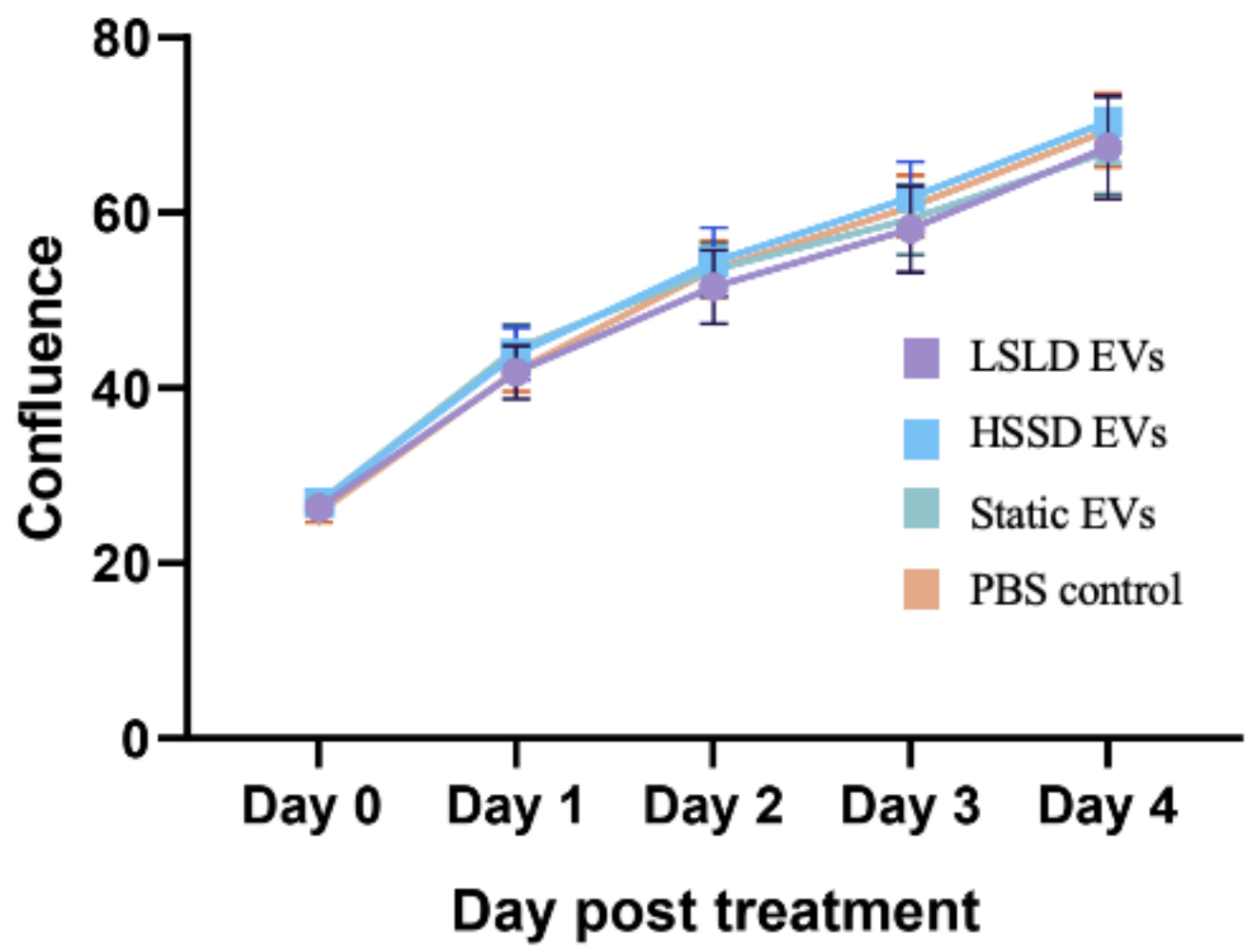

Supplemental Figure 7: Mechanical strain does not alter the effects of primary myogenic cell-derived EVs on proliferation of primary myogenic cells. C57BL6 Primary **myogenic cells** (P10) were seeded at 10,000 cells per well in 1 mL growth media in a 24-well-plate, to achieve 20% confluency on Day 0 (at the time of EV treatment). Wells were treated with 1e10 EVs/mL of HSSD EVs, LSLD EVs, Static EVs, or PBS alone and cell confluence was monitored every 24 hours over 4 days using IncuCyte live cell imaging. No significant differences in cell confluence were observed between treatment groups at any timepoint. Statistical comparisons were performed using a one-way ANOVA at each timepoint.
